# PLK1/vimentin signaling facilitates immune escape by recruiting Smad2/3 to PD-L1 promoter in metastatic lung adenocarcinoma

**DOI:** 10.1038/s41418-021-00781-4

**Published:** 2021-05-07

**Authors:** Hay-Ran Jang, Sol-Bi Shin, Chang-Hyeon Kim, Jae-Yeon Won, Rong Xu, Da-Eun Kim, Hyungshin Yim

**Affiliations:** 1 Department of Pharmacy, College of Pharmacy, Hanyang University, Ansan, Gyeonggi-do Korea; 2 Institute of Pharmaceutical Science and Technology, Hanyang University, Ansan, Gyeonggi-do Korea

**Keywords:** Cancer microenvironment, Prognostic markers

## Abstract

The prerequisite function of vimentin for the epithelial–mesenchymal transition (EMT) is not clearly elucidated yet. Here, we show that vimentin phosphorylated by PLK1, triggers TGF-β-signaling, which consequently leads to metastasis and PD-L1 expression for immune suppression in lung adenocarcinoma. The clinical correlation between expression of both vimentin and PLK1, and overall survival rates of patients was significant in lung adenocarcinoma but not in squamous cell carcinoma. The phosphorylation of vimentin was accompanied by the activation of PLK1 during TGF-β-induced EMT in lung adenocarcinoma. Among the several phosphorylation sites determined by phospho-proteomic analysis and the site-specific mutagenesis, the phosphorylation at S339 displayed the most effective metastasis and tumourigenesis with the highest expression of PD-L1, compared with that of wild-type and other versions in both 3D cell culture and tail-vein injection metastasis models. Phosphomimetic vimentin at S339 interacted with p-Smad2 for its nuclear localization, leading to the expression of PD-L1. Clinical relevance revealed the inverse correlation between the survival rates of patients and the expressions of *VIM, PLK1*, and *CD274* in primary and metastatic lung adenocarcinoma. Thus, PLK1-mediated phosphorylation of vimentin activates TGF-β signaling pathway, leading to the metastasis and immune escape through the expression of PD-L1, functioning as a shuttling protein in lung adenocarcinoma.

## Introduction

Lung adenocarcinoma (LUAD) is one of the most aggressive and lethal cancers, which accounts for ≈40% of all lung cancers, with average overall survival (OS) of less than 5 years, as it is often diagnosed at advanced stages [1]. According to recent data (https://seer.cancer.gov/statfacts/), metastasis rather than the primary tumors is the major cause of death in cancer patients [2, 3]. In the early stages of metastasis, epithelial cells undergo changes in morphogenesis and activity in a process called the epithelial–mesenchymal transition (EMT), which is critical in metastatic progress [2, 4]. Through genetic reprogramming, cells gain mesenchymal characteristics, and increased levels of N-cadherin and vimentin result from activation of mesenchymal transcriptional factors [5].

Vimentin, ubiquitously expressed in mesenchymal and mesenchymal-derived cells in adults [6], maintains cellular integrity as a main component of intermediate filaments (IFs) [7], is found in focal adhesions, which are dynamic protein complexes interlinking cytoskeleton with extracellular matrix (ECM), and contributes to cell migration and invasion during EMT [8–13]. High vimentin levels indicate poor prognosis in several carcinomas, including breast [14], lung [15], and stomach [16], because they correlate with invasiveness [17, 18] and EMT [10, 19]. Previous studies showed that vimentin knockout reduced mechanic motility in embryonic cells in vitro and impaired the wound healing in vimentin-deficient mice [8, 9, 20, 21], indicating vimentin is required for mechanical cell migration during metastasis. How the vimentin network regulates tumor metastasis and lung cancer survival remains unclear. Phosphorylation of vimentin is associated with the regulation of IF structure and cell motility [22, 23], and increased motility likely results from the phosphorylation-induced disassembly of vimentin IFs. Upon activation of AKT1, phosphorylation of vimentin at Ser39 protects against caspase-mediated proteolysis in sarcomas metastasis [23]. In cancer invasion, PLK1 is also associated with phosphorylation of vimentin at Ser83, which regulates cell surface β1-integrin [24], though its regulatory mechanism in metastasis remains unclear.

PLK1 overexpression induces EMT, increasing cell motility in non-small cell lung cancer (NSCLC) [25], prostate cancer [26], and gastric cancer [27]. Catalytically active PLK1 promotes metastasis by activating TGF-β signaling, which increases factors related to ECM, focal adhesions, and adherens junctions in the transcriptome profile [25]. Because vimentin is found at focal adhesions, we sought to investigate the relationship between PLK1-driven metastasis and vimentin function, along with contributions of the vimentin network to metastasis. To explore this, we analyzed patient survival based on the expression of PLK1 and vimentin in both LUAD and lung squamous cell carcinoma (LUSQ). Here, we demonstrate that in LUAD, PLK1-mediated phosphorylation of vimentin accelerates metastasis and immune escape from cytotoxic T cells through TGF-β signaling and recruitment of p-Smad2/3 to the PD-L1 promoter.

## Materials and methods

### Materials

Minimum essential medium (MEM), RPMI 1640 medium, Dulbecco’s modified Eagle’s medium (DMEM), fetal bovine serum (FBS), penicillin, and streptomycin were purchased from Corning Cellgro (Manassas, VA, USA). Transforming growth factor (TGF)-β and all other chemical reagents were purchased from Sigma-Aldrich (St. Louis, MO, USA). Withaferin A, niclosamide, SB431542, and U0126 were obtained from Chromadex (Irvine, CA, USA), Selleck Chemicals (Houston, TX, USA), Tocris Bioscience (Bristol, UK), and Cell Signaling Technology (Beverly, MA, USA), respectively.

### Cell culture and treatment

The A549 and NCI-H460 cell lines were purchased from KCLB (KCLB; Seoul, Korea) and HEK293T cells were from ATCC (ATCC; Manassas, VA, USA). HEK293T, NCI-H460, and A549 cells were authenticated by STR profiling, tested for mycoplasma contamination, and grown in RPMI 1640, DMEM, MEM (Invitrogen; Carlsbad, CA, USA), supplemented with 10% FBS in the presence of antibiotics in a humidified 5% CO_2_ incubator at 37 °C. For the administration of TGF-β, cells were seeded at 1 × 10^5^ cells/ml, and 16 hours later, the cells were treated with 2.5 ng/ml of TGF-β for 48 hours.

### Quantitative reverse transcription polymerase chain reaction (qRT-PCR)

Total RNA was extracted 48 h after exposure to TGF-β and quantified by Nanodrop (Thermo Scientific; Wilmington, DE, USA). Next, cDNA was generated with a First Strand cDNA Synthesis Kit (Thermo Scientific). After the synthesized cDNA was mixed with SYBR Green Master Mix (Bio-Rad; Hercules, CA, USA) and various sets of gene-specific primers, qRT-PCR was performed using a CFX96 Real-Time PCR system (Bio-Rad). The primer sequences used are shown in Supplementary Table 1.

### Immunoblot analysis

Cells were lysed in lysis buffer [0.5% Triton X-100, 20 mM Tris (pH 7.5), 2 mM MgCl_2_, 1 mM dithiothreitol (DTT), 1 mM EGTA, 50 mM β-glycerophosphate, 25 mM NaF, 1 mM Na vanadate, 100 mg/ml PMSF, and protease inhibitor cocktail (Roche; Indianapolis, IN, USA)]. After adjusting the protein concentration, proteins were resolved by SDS-PAGE and subjected to immunoblot analysis with the appropriate antibodies as follows: vimentin (Santa Cruz Biotechnology, sc-7557); phospho-vimentin^S82^ (MBL, D095-3); PLK1 (Millipore, 05-844); phospho-PLK1^T210^ (Cell Signaling, 5472); N-cadherin (Sigma, C3865); E-cadherin (Cell Signaling, #4065); PD-L1 (Cell Signaling, 13684); PD-L2 (Invitrogen, PA5-20344); Smad2/3 (Cell Signaling, 8685); phospho-Samd2^S465/S467^ (Cell Signaling, 18338); Stat3 (Santa Cruz Biotechnology, sc-8019); phospho-Stat3^T705^ (Santa Cruz Biotechnology, sc-7993); c-Jun (Santa Cruz Biotechnology, sc-74543), c-fos (Santa Cruz Biotechnology, sc-52), NF-kB^p65^ (Santa Cruz Biotechnology, sc-71677), Erk1/2 (Cell Signaling, 4695), phospho-Erk1/2^T202/Y204^ (Cell Signaling, 4370), Histone H1 (Santa Cruz Biotechnology, sc-8030), GAPDH (Sigma, G8795), β-actin (Sigma, A5441); and IgG (Santa Cruz Biotechnology, sc-2027). Immune complexes were revealed using an Odyssey infrared imaging system (LI-COR Biosciences; Lincoln, NE, USA). Intensity values were determined using LI-COR Odyssey software.

### Wound-healing assay

NCI-460 cells were seeded at 2 × 10^5^ cells/ml, and a wound was established by scratching one time with a 1 mm thick pipette tip. Wounded monolayer images were collected and analysed with an Eclipse Ti microscope (Nikon, Tokyo, Japan) at the indicated times.

### Transwell cell migration and inverted invasion assay

Transwell cell migration and invasion assays were performed as described previously [25]. Briefly, cell migration assays were conducted using 24-well plates with 8-μm pore Transwell chambers (Corning, NY, USA). The lower chamber was filled with culture medium containing 10% FBS. NCI-H460 cells were suspended at a density of 5 × 10^4^ cells/well in RPMI medium, without FBS and added to the upper chamber. Three days after seeding, the cells on the bottom layer surface were stained with 0.05% crystal violet dye, and the intensity values were measured using an Odyssey infrared imaging system (LI-COR Biosciences). For the cell invasion assay, cells were seeded at a density of 1 × 10^5^ cells/well in the upper chamber filled with Matrigel (BD Biosciences, Erembodegem, Belgium). Five to seven days after seeding, the cells on the bottom layer surface were stained with 0.05% crystal violet dye, and after treatment with DMSO, the absorbance was measured at 590 nm using an M4 microplate reader (Molecular Devices, CA, USA).

### Colony formation assay

The colony formation assay was performed as described previously [25]. In total, 1 × 10^3^ cells were resuspended in 1 ml of medium with 10% FBS in 0.4% agar and overlaid onto a bottom agar layer composed of 10% FBS and 0.6% agar in 1 ml of medium in a 12-well plate. The cells were incubated in a humidified 5% CO_2_ incubator at 37 °C. After 2 weeks, the colonies formed in the agar were counted after staining with 0.005% crystal violet.

### PLK1 kinase assay

For the expression and purification of PLK1 kinase, a baculovirus encoding a GST-tagged T210D mutant of PLK1, a constitutively active form of PLK1, was used to infect Sf9 and Hi5 insect cells, as described previously [28]. Glutathione S-transferase (GST)-tagged PLK1 protein was purified using glutathione-Sepharose 4B beads (GE Healthcare Life Sciences), as stated in the manufacturer’s instructions. The purified active PLK1 kinase was used with GST-tagged vimentin as a substrate of PLK1 in the in vitro PLK1 kinase assay, which was performed in reaction buffer containing 25 μM ATP and 10 μCi [γ-^32^P] ATP at 37 °C for 30 min. A GST-fused TCTP protein was used as a positive control. The reacted samples were suspended in SDS loading buffer, resolved by SDS-PAGE, and detected by autoradiography.

### Immunoprecipitation assay

Lysates of A549 and NCI-H460 cells were incubated with normal IgG (Santa Cruz Biotechnology) and anti-PLK1 (Millipore, 05-844) antibodies for 16 h at 4 °C with end-over-end mixing, followed by incubation with protein A/G agarose (Santa Cruz Biotechnology) for 2 h at 4 °C. Immunoprecipitants were separated from the supernatants by centrifugation and washed four times with lysis buffer. Proteins were resolved by SDS-PAGE and analysed by immunoblot.

### In-gel digestion with trypsin and extraction of peptides

A PLK1 kinase assay was performed using the purified active form of PLK1 (T210D) and GST-tagged vimentin as a substrate for PLK1 in reaction buffer containing cold 25 μM ATP at 37 °C for 30 min. The phosphorylated GST-vimentin proteins were resolved by SDS-PAGE, and the band from the SDS-PAGE was in-gel digested with trypsin. After incubation in 25 mM ammonium bicarbonate buffer, pH 7.8, at 37 °C overnight, the tryptic peptides were extracted with 5 μl of 0.5% TFA containing 50% (v/v) ACN for 40 min with mild sonication. The extracted solution was concentrated using a centrifugal vacuum concentrator. Prior to mass spectrometric analysis, the peptide solution was subjected to a desalting process using a reversed-phase column. The bound peptides were eluted with 5 μl of 70% ACN and 5% (v/v) formic acid.

### LC–MS/MS analysis

A nano LC-MS/MS analysis was performed with an Easy n-LC (Thermo Fisher, San Jose, CA, USA) and an LTQ Orbitrap XL mass spectrometer (Thermo Fisher, San Jose, CA, USA) equipped with a nano-electrospray source. Samples were separated on a C18 nano-bore column (150 mm × 0.1 mm, 3 μm pore size; Agilent). Mobile phase A for the LC separation was 0.1% formic acid and 3% acetonitrile in deionized water, and mobile phase B was 0.1% formic acid in acetonitrile. The chromatography gradient was designed for a linear increase from 5% B to 55% B in 40 min, 52% B to 75% B in 4 min, 95% B in 4 min, and 3% B in 6 min. The flow rate was maintained at 1500 nL/min. Mass spectra were acquired using data-dependent acquisition with a full mass scan (350–1200 m/z) followed by 10 MS/MS scans. For the MS1 full scans, the orbitrap resolution was 15,000, and the AGC was 2 × 10^5^. For MS/MS in the LTQ Orbitrap XL, the AGC was 1 × 10^4^. The individual spectra from the MS/MS were processed using SEQUEST software (Thermo Quest, San Jose, CA, USA), and the generated peak lists were used to query using the MASCOT program (Matrix Science Ltd., London, UK). We set the tolerance of the peptide mass to 10 ppm. The MS/MS ion mass tolerance was 0.8 Da, allowance of missed cleavage was 2, and charge states (+2 and +3) were taken into account for data analysis. We took only significant hits as defined by a MASCOT probability analysis.

### Generation of phosphomimetic and non-phosphomimetic mutant of vimentin

Using plasmids of GST-tagged mouse vimentin (gene access no. NM_ 011701), the putative phosphorylation sites of vimentin were converted to Ala using each mutagenesis primer. Mutagenesis was performed using a QuickChange II Site-Directed Mutagenesis Kit (Promega; Madison, WI, USA) according to the manufacturer’s protocol. The primer sequences used for converting to Ala or Glu are shown in Supplementary Table 2.

### Lentivirus-based plasmid preparation, virus production, and infection

Lentivirus-based RNAi transfer plasmids targeting human vimentin (gene access no. NM_003380) at positions 1447–1467 (GTGAAATGGAAGAGAACTTTG) (pLKO-Puro.1-vimentin) were prepared, and lentivirus was generated as described previously [25, 29]. For the expression of mouse vimentin, pLVX-TRE3G-eRFP-mVimentin and pLVX-Tet3G vectors (Clontech #631351; Palo Alto, CA, USA) were used. Wild-type and mutant vimentin were amplified using a forward primer (5′-ACGGGGCCCATGTCCACCAGGTCCGTGTC-3′) and a reverse primer (5′-ACGACGCGTTTATTCAAGGTCATCGTGA-3′), subcloned into pLVX-TRE3G-eRFP using the ApaI and MluI restriction enzymes, and expressed using the lentivirus, as described previously [25].

### Experimental lung metastasis assay

Four-week-old male BALB/c nude mice (Orient Bio, Seoul, Korea) were injected with NCI-H460 cells stably expressing pLVX-TRE3G-eRFP-Tet3G-Mock, wild-type (WT) vimentin or phospho-mutant S339A, S339E, T327A, T327E, S83A, or S83E vimentin (2 × 10^6^ cells/100μl phosphate buffered saline) via the tail vein. The mice received 1 mg/ml of doxycycline in their drinking water to induce TRE3G-vimentin overexpression. Eight weeks after injection, all mice were sacrificed, and their lungs were separated and fixed in 4% paraformaldehyde for H&E staining and Ki67 tissue staining. All animal experiments were approved and managed by the guidelines of the Institutional Animal Care and Use Committee, Hanyang University (HY-IACUC-2017-0115A).

### Bioinformatics analysis

NSCLC patient data were obtained from an online database (www.kmplot.com) following a previous report [30]. All cancer patients in the database were identified from the Cancer Biomedical Informatics Grid (http://cabig.cancer.gov/, microarray samples are published by the caArray project), the Gene Expression Omnibus (http://www.ncbi.nlm.nih.gov/geo/), or the Cancer Genome Atlas (http://cancergenome.nih.gov). The database, which was established using gene expression data and survival information from 1926 NSCLC patients, was used to establish the clinical relevance of vimentin expression to the survival times of NSCLC patients, after excluding biased arrays. The expression values for vimentin and clinical data from those samples were extracted and used for the survival analysis. The samples were split into high and low groups using vimentin expression. Hazard ratios (HR) with 95% confidence intervals (CI) and the log rank *P* were calculated according to the formulas on each database’s webpage. A *p* value of <0.05 was considered to be statistically significant. An HR is the ratio of the hazard rates that correspond to the conditions described by two levels of an explanatory variable in survival analysis.

### Transcriptome profiling

RNA was extracted from the indicated cells expressing mock, wild-type, phosphomimetic (S339E), or non-phosphomimetic vimentin (S339A). RNA purity and integrity were evaluated using an ND-1000 spectrophotometer (Nanodrop, Wilmington, DE, USA) and Agilent 2100 bioanalyzer (Agilent Technologies, Palo Alto, CA, USA). The Affymetrix whole transcript expression array process was executed according to the manufacturer’s protocol (GeneChip Whole Transcript PLUS reagent kit). cDNA was synthesized using a GeneChip WT (Whole Transcript) Amplification kit as described by the manufacturer. The sense cDNA was then fragmented and biotin-labeled with terminal deoxynucleotidyl transferase using a GeneChip WT Terminal labeling kit. Approximately 5.5 μg of labeled DNA target was hybridized to the Affymetrix GeneChip Human 2.0 ST Array at 45 °C for 16 hours. Hybridized arrays were washed and stained on a GeneChip Fluidics Station 450 and scanned on a GCS3000 Scanner (Affymetrix). Signal values were computed using Affymetrix® GeneChip™ Command Console software.

### Microarray analysis

Raw data were extracted automatically using an Affymetrix data extraction protocol in the Affymetrix GeneChip® Command Console® software. After importing CEL files, the data were summarized and normalized with the robust multi-average (RMA) method implemented in the Affymetrix® Expression Console™ software. We exported the results of the gene-level RMA analysis and performed a differentially expressed gene analysis. The analysis comparing the wild-type PLK1 or constitutive active PLK1 with the non-invasive mock was carried out using fold changes. For transcriptome data, gene probes with significant fold changes (more than 1.5) were clustered. To develop a significant probe list, we performed a gene-enrichment and functional annotation analysis using gene ontology (http://geneontology.org/) and KEGG (http://kegg.jp). All statistical tests and visualizations of differentially expressed genes were conducted using R statistical language v. 3.1.2. (www.r-project.org).

### Chromatin immunoprecipitation assays

The ChIP assay was performed as described [31] with slight modification. To examine the interaction between Smad2/3 or STAT3 and the PD-L1 promoter, NCI-H460 cells expressing wild-type, S339A, or S339E vimentin were used. Cross-linking was done with 1.4% formaldehyde. Cells were lysed with IP buffer [0.5% Triton X-100, 20 mM Tris, pH 7.5, 2 mM MgCl_2_, 1 mM DTT, 1 mM EGTA, 50 mM β-glycerophosphate, 25 mM NaF, 1 mM Na vanadate, 100 μg/ml PMSF, and protease inhibitor cocktail (Roche; Indianapolis, IN, USA)]. Chromatin was sheared by sonication and incubated with polyclonal antibodies to Smad2/3 or normal IgG for 16 hours. Sheared chromatin was incubated with protein A/G beads (Santa Cruz; CA, USA) for 2 hours and washed five times with IP buffer. Chelex 100 slurry (Bio-Rad; Hercules, CA, USA) was added to the washed beads, which were then boiled and incubated with Proteinase K (Invitrogen; Carlsbad, CA, USA) at 55 °C for 30 min. The samples were boiled again and cleared by centrifugation, and then the supernatants were taken for real-time PCR. The bound chromatin fraction was amplified with human PD-L1 promoter-specific primers flanking CAGA boxes (Smad2/3 binding sites) at –554 to –551, –443 to –440, and –371 to –368 for 40 cycles. The forward primer was 5ʹ-CTTAATCCTTAGGGTGGCAGA-3ʹ, and the reverse primer was 5ʹ-AGGCGTCCCCCTTTCTGA-3ʹ. For *STAT3* binding sites, the bound chromatin fraction was amplified with human PD-L1 promoter-specific primers flanking TTCC(G = C)GGAA at –212 to –202, –134 to –120 for 40 cycles. The forward primer was 5ʹ-CAAGGTGCGTTCAGATGTTG-3ʹ, and the reverse primer was 5ʹ-GGCGTTGGACTTTCCTGA-3ʹ. Real time PCR was carried out on a CFX96 Real-Time PCR system (Bio-Rad) using SYBR Green Master Mix (Bio-Rad, #1708880). The data were analysed by the comparative C_T_(ΔΔC_T_) method. The relative occupancy of the immunoprecipitated factor at a locus was estimated using the following equation: 2^(Ct^IgG^−Ct^smad2/3^), where Ct^IgG^ and Ct^smad2/3^ are the mean threshold cycles of PCR done in triplicate on DNA samples from normal IgG and Smad2/3 immunoprecipitations, respectively.

### Statistical analysis

All data are given as means ± SDs of at least three independent experiments, each performed in triplicate. Results were analysed for statistically significant differences using the student’s *t*-test, and statistical significance was set at *p* < 0.01: *; *p* < 0.05. **; *p* < 0.01. ***; *p* < 0.001.

## Results

### Clinical relevance of active PLK1 and vimentin in metastatic LUAD

**Fig. 1:**
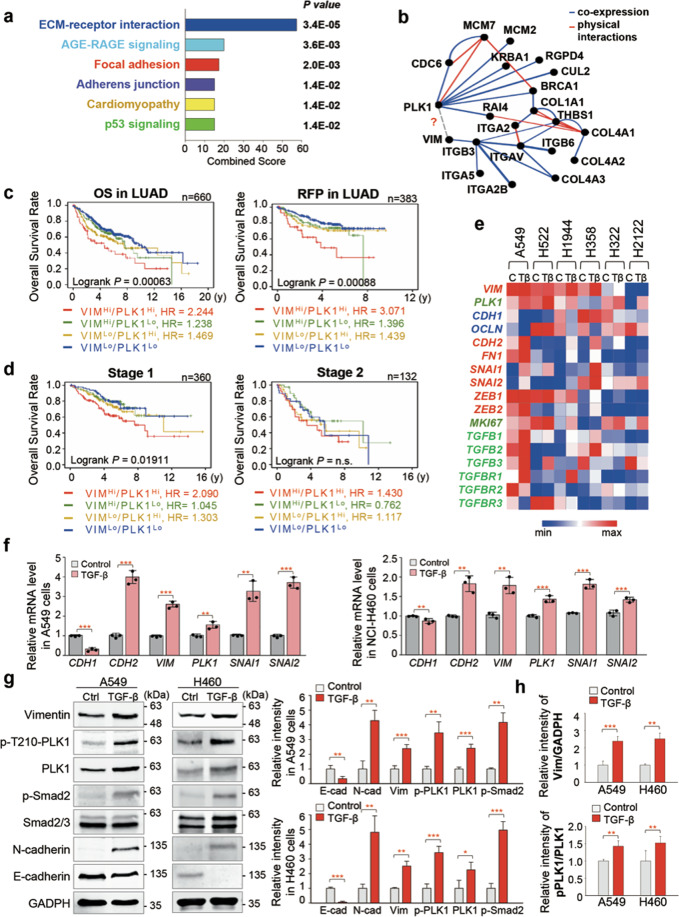
The correlation between active PLK1 and vimentin expression during epithelial-mesenchymal transition in lung adenocarcinoma. **a** Analysis of transcriptome data for gene probes with fold changes of more than 1.5 (general cut-off point for DEGs) and a combined score calculated by multiplying the ln (*p* value) and *z*-score among the invasive cells expressing active PLK1 during EMT of A549 cells. The significant genes were categorized using the KEGG 2019 pathway. **b** Analysis of the transcriptome data for gene probes in the invasive cells expressing active PLK1 during EMT of A549 cells. The network of ECM factors was extracted from the GeneMANIA database. **c** The overall survival (OS) times (left panel, *n* = 660) or the relapse free progression (RFP) rates (right panel, *n* = 383) of patients with lung adenocarcinoma were analysed according to their *PLK1* and *VIM* expression levels. High (Hi) *vs*. low (Lo) expression was split by auto cut off value of KM PLOTTER. y; years. **d** Cumulative OS times of lung adenocarcinoma patients with stage 1 (left panel, *n* =360), or stage 2 (right panel, *n* = 132) were generated by splitting patients according to their expression of *PLK1* and *VIM*. High (Hi) *vs*. low (Lo) expression was split by mean value. n.s., not significant. **e** A heatmap analysis was performed for *VIM, PLK1*, epithelial markers *CDH1* and *OCLN*, and several mesenchymal markers, including *CDH2*, using a published transcriptome of TGF-β-treated NSCLC (GSE114761). **f** QRT-PCR was performed for *CDH1, CDH2*, *VIM, PLK1*, *SNAI1*, and *SNAI2* expression using A549 (left panel) and NCI-H460 (right panel) lung adenocarcinoma cells treated with 2.5 ng/ml of TGF-β for 48 hours. *p < 0.05; **p < 0.01; ***p < 0.001; (*n* = 3). Data are presented as mean ± SD. **g** Immunoblotting was performed to measure the expression and phosphorylation of PLK1 using anti-PLK1 and anti-p-PLK1 (T210) antibodies in A549 and NCI-H460 cells. The band intensity values of E-cadherin, N-cadherin, vimentin, p-Samd2^S465/S467^, p-PLK1^T210^, and PLK1 were quantified using LI-COR Odyssey software (Li-COR Biosciences). **h** The relative intensity values of p-T210-PLK1 and vimentin were normalized to those of PLK1 and GAPDH, respectively. All experiments were performed at least three independent experiments.

During PLK1-driven EMT, changes in genes related to ECM and focal adhesion were ranked within the top 3 by KEGG pathway analysis using our recent data (Fig. 1a) [25]. Vimentin is a main component of IFs and is found in focal adhesions [10–12] and could be a substrate of PLK1 during cancer invasion [24]. However, the network extracted from the GeneMANIA database [32] showed unclear interaction between vimentin and PLK1 (Fig. 1b). We decided to investigate the vimentin contribution to EMT in active PLK1–driven metastasis of NSCLC.

The clinical relationship between PLK1 or vimentin and cumulative OS in lung cancer was analysed using KM PLOTTER [30] (Fig. 1c, d; Supplementary Fig. 1; Supplementary Tables 3–4). OS or relapse-free progression (RFP) survival of LUAD patients with high *PLK1/VIM* expression were significantly shorter than those with low *PLK1/VIM* expression (left, *n* = 660, HR = 2.244, log rank *P* = 0.00063; right, *n* = 383, HR = 3.071, log rank *P* = 0.00088) (Fig. 1c, Supplementary Table 3). In LUSQ, however, OS rates of patients with high *VIM* expression were longer than those with low *VIM* expression (Supplementary Fig. 1). In a clinical analysis of 360 adenocarcinoma patients with stage 1 (Fig. 1d, left panel, Supplementary Table 4), those with high levels of *PLK1/VIM* had shorter OS than those with low *PLK1/VIM* levels (*n* = 360, HR = 2.090, log rank *P* = 0.019). Similar results were seen among stage 2 LUAD patients (*n* = 132, HR = 1.430, log rank *P* = 0.3725) (Fig. 1d, right panel). Thus, high expression of both vimentin and PLK1 could be used as a negative index for survival in primary and metastatic LUAD, but not in LUSQ.

To observe expression of *VIM* and *PLK1* in metastatic LUAD, we analysed a published dataset (GSE 114761) [33] (Fig. 1e; Supplementary Table 5). Adenocarcinoma cells without EMT were ruled out under the condition of TGF-β treatment, as determined by changes in EMT markers. Heatmap analysis revealed upregulated *VIM* and *PLK1* mRNA expression in TGF-β-treated adenocarcinoma when the mesenchymal markers *CDH2*, *SNAI1*, and *SNAI2* were high and either *CDH1* or *OCLN* (epithelial markers) was low in majority of LUAD cells analysed (Fig. 1e).

To functionally assess the effects of vimentin and PLK1 on TGF-β-induced EMT, we performed qRT-PCR and immunoblotting in TGF-β−treated A549 and NCI-H460 cells (Fig. 1f–h). TGF-β treatment increased levels of *VIM* and *PLK1* with lower *CDH1* and higher *CDH2* expression compared with the control (Fig. 1f). Changes in vimentin, PLK1, E-cadherin, and N-cadherin protein were similar to those of the mRNA (Fig. 1g). In addition, TGF-β treatment increased the level of active PLK1 phosphorylated at T210 with p-Smad2 in TGF-β-treated cells compared with the control (Fig. 1g, h). Thus, levels of active PLK1 and vimentin increased with TGF-β-induced EMT in LUAD cells.

### Phosphorylation of vimentin through direct interaction with PLK1 in TGF-β-treated LUAD cells

**Fig. 2:**
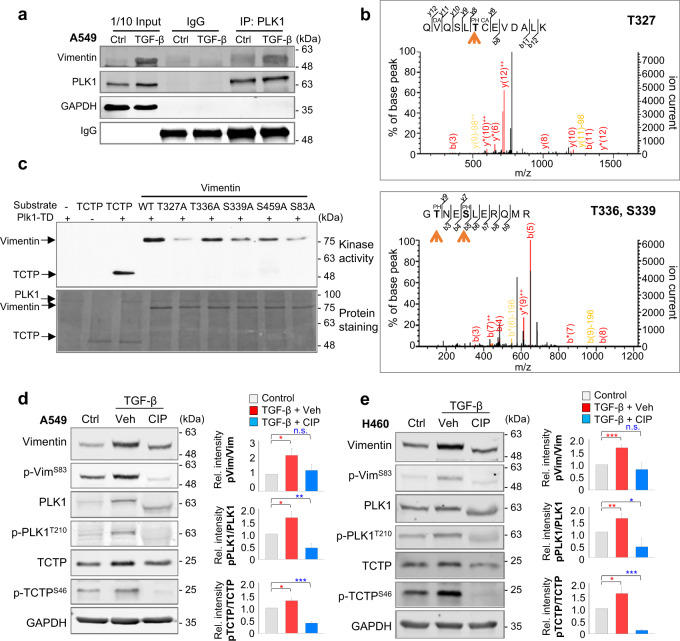
Vimentin is phosphorylated by PLK1 at T327, S339, S459, and S83 in vitro. **a** A549 cells were treated with 2.5 ng/ml of TGF-β for 48 hours to induce EMT. Immunoprecipitation of cell lysates was performed with anti-PLK1 antibody or normal IgG followed by immunoblotting with anti-vimentin antibody. **b** Possible sites on vimentin phosphorylated by PLK1 were newly detected in the LC-MS/MS analysis at the T327, T336, and S339 residues. **c** Purified GST-tagged wild-type, T327A, T336A, S339A, S459A, and S83A vimentin mutants were used for a PLK1 kinase assay with radioactive ATP. **d**,**e** Phosphorylation of vimentin occurs in A549 (**d**) and NCI-H460 (**e**) cells treated with 2.5 ng/ml of TGF-β for 48 hours. Treatment with phosphatase (CIP) reduced the phosphorylational modification of vimentin and PLK1 in TGF-β-induced EMT. TCTP was used as a positive control of the PLK1 substrate. All experiments were performed at least three independent experiments. *p < 0.05; **p < 0.01; ***p < 0.001; (*n* = 3). n.s. not significant.

Previously, we revealed that active PLK1 drives metastasis of NSCLC through expression of catalytic mutants of PLK1 and inhibition of PLK1 activity using PLK1-specific inhibitors or shRNA [25]. Since active PLK1 and vimentin were high in TGF-β-treated LUAD (Fig. 1e–h), we investigated whether vimentin is regulated by PLK1 in TGF-β-induced metastatic LUAD. We observed the interaction between PLK1 and vimentin in TGF-β-treated LUAD cells. Coimmunoprecipitation showed that endogenous PLK1 and vimentin interacted during TGF-β-induced EMT (Fig. 2a; Supplementary Fig. 2a) even under normal conditions.

To examine whether PLK1 phosphorylates vimentin during EMT, we performed an in vitro PLK1 kinase assay, which showed phosphorylation of vimentin by PLK1 (Supplementary Fig. 2b). To explore phosphorylation sites of vimentin by PLK1, LC-MS/MS analysis was performed (Fig. 2b), and revealed newly detected potential phosphorylation sites at the T327, T336, and S339 residues. Because previous studies reported that S83 and S459 residues were phosphorylation sites of PLK1 in mitosis [34, 35], we used site-directed mutagenesis to replace those residues and the newly detected residues with non-phosphomimetic alanine and compared the phosphorylation ratios. PLK1 kinase assay found that phosphorylation of alanine mutants at T327, S339, S83, and S459 was markedly reduced, but T336A was not (Fig. 2c), indicating that PLK1 phosphorylates vimentin at T327, S339, S459, and S83 in vitro. To observe whether vimentin phosphorylation depends on cell cycle or EMT, NCI-H460 cells were treated with hydroxyurea, nocodazole, or TGF-β. Phosphorylation of vimentin at S83 increased in TGF-β-induced EMT as well as mitosis compared with the control (Supplementary Fig. 2c). Phosphatase treatment reduced p-vimentin^S83^ and p-PLK1^T210^ upregulation by TGF-β treatment, indicating that phosphorylation of vimentin by PLK1 occurs during EMT (Fig. 2d, e). The residues are evolutionarily conserved in several species (Supplementary Fig. 2d), implying their importance to the physiological regulation of vimentin.

### Phosphorylation of vimentin by PLK1 accelerates cell motility and invasiveness

**Fig. 3:**
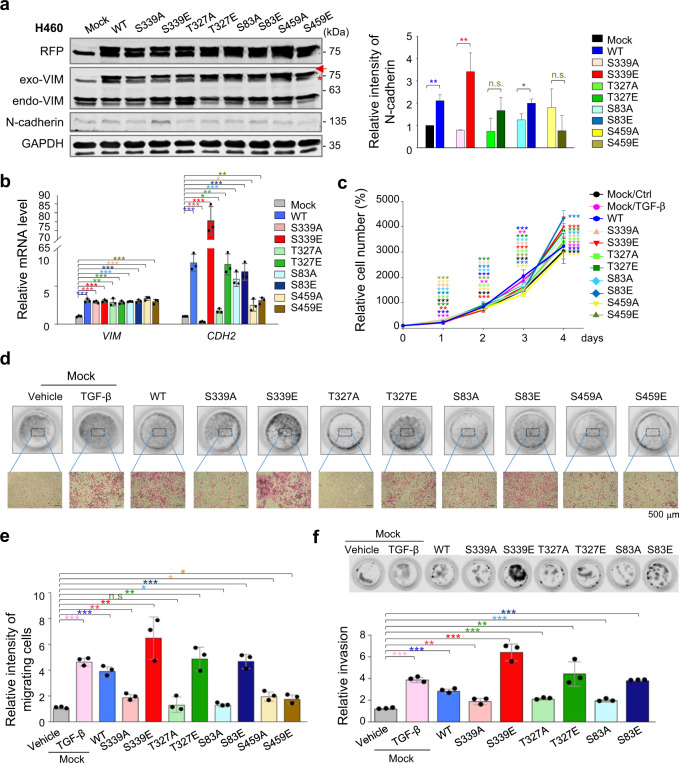
Phosphorylation of vimentin by PLK1 at S339, T327, and S83 accelerates cancer-cell motility and invasiveness. RFP-tagged empty vector (Mock), wild-type (WT) vimentin and S339A, S339E, T327A, T327E, S83A, S83E, S459A, and S459E mutants were expressed in NCI-H460 lung adenocarcinoma cells. **a** NCI-H460 cells were treated with doxycycline for 48 hours to express RFP-tagged vimentin. Immunoblotting was performed using anti-RFP, anti-vimentin, anti-N-cadherin, and anti-GAPDH (left panel). Endo-VIM, endogenous vimentin; Exo-VIM, exogenous vimentin. The band intensity values of N-cadherin were quantified using LI-COR Odyssey software (Li-COR Biosciences), normalized, and plotted (right panel). *p < 0.05; **p < 0.01; (*n* = 3). n.s., not significant. Arrow, exogenous vimentin band; *, non-specific band. **b** QRT-PCR was performed for *CDH2* and *VIM* in NCI-H460 cells expressing various versions of vimentin. *p < 0.05; **p < 0.01; ***p < 0.001; (*n* = 3). Data are presented as mean ± SD. **c** Cell proliferation assay was performed (*n* = 3). *p < 0.05; **p < 0.01; ***p < 0.001; (*n* = 3). Data are presented as mean ± SD. **d** Cells expressing wild-type or mutants of vimentin were subjected to a Transwell migration assay. As a positive control for migration, cells were treated with TGF-β. Three days after seeding, the cells on the bottom layer surface were stained with 0.05% crystal violet dye. Images of the Transwell cell migration assay were collected and analysed with a microscope. Bar scale, 500 μm. **e** The intensity values of the stained cells in the Transwell cell migration assay were measured using an Odyssey infrared imaging system (LI-COR Biosciences) and plotted. *p < 0.05; **p < 0.01; ***p < 0.001; (*n* = 3). Data are presented as mean±SD. **f** An invasion assay was performed using NCI-H460 cells expressing wild-type or mutants of vimentin. Five days after seeding, the cells that invaded the bottom layer surface were stained with 0.05% crystal violet dye, and the relative absorbance was plotted (*n* = 3). All experiments were performed at least three independent experiments. Data are presented as mean±SD of three independent experiments (significantly different as compared with experimental control). n.s. no significant.

Because PLK1 phosphorylates vimentin through direct interaction in TGF-β-treated cells (Fig. 2), we next investigated the effects of p-vimentin on cell motility and invasion. Vimentin phosphorylated or unphosphorylated at S339, T327, S83, or S459 was expressed using a lentiviral doxycycline-inducible system, showing the levels of exogenous vimentin were similar to those of endogenous proteins (Fig. 3a). mRNA and protein levels of N-cadherin increased markedly in cells expressing S339E compared with those without phosphorylation (Fig. 3a, b; Supplementary Fig. 3). The level of endogenous vimentin was not affected by that of the exogenous form, probably due to saturation. The effects on cell proliferation were observed (Fig. 3c). The proliferation of cells expressing S83E, S339E, and T327E was greater than that of cells expressing non-phosphomimetic vimentin. In addition, motility of cells expressing S339E, T327E, and S83E increased compared with that of those expressing wild-type and those treated with TGF-β as a positive control (Fig. 3d–e), as determined by a Transwell migration assay. Motility of S339E increased dramatically, by 6-fold compared with the mock control, when the relative motility of the control was defined as 1 (Fig. 3e). Motility of cells expressing T327E and S83E was ≈5-fold higher than that of mock controls. Thus, phosphorylation of vimentin by PLK1 at S339, T327, and S83 promoted cell motility compared with non-phosphomimetics. Because S459E did not affect cell motility or N-cadherin level, S459 residue was ruled out for further study.

To evaluate whether the phosphomimetics triggered invasiveness, we performed invasion assay (Fig. 3f). The highest absorbance was shown in NCI-H460 cells expressing phosphomimetics at S339, and the next highest in cells expressing T327E and S83E (Fig. 3f), indicating that the phosphorylation of vimentin at S339, T327, and S83 promotes cancer invasiveness and cell motility. Of note, cells expressing S339E had higher EMT production, migration, and invasion abilities than cells expressing T327E, S83E, or wild-type.

### Phosphorylation of vimentin at S339 accelerates tumorigenesis by expressing PD-L1 in metastatic cancer

**Fig. 4:**
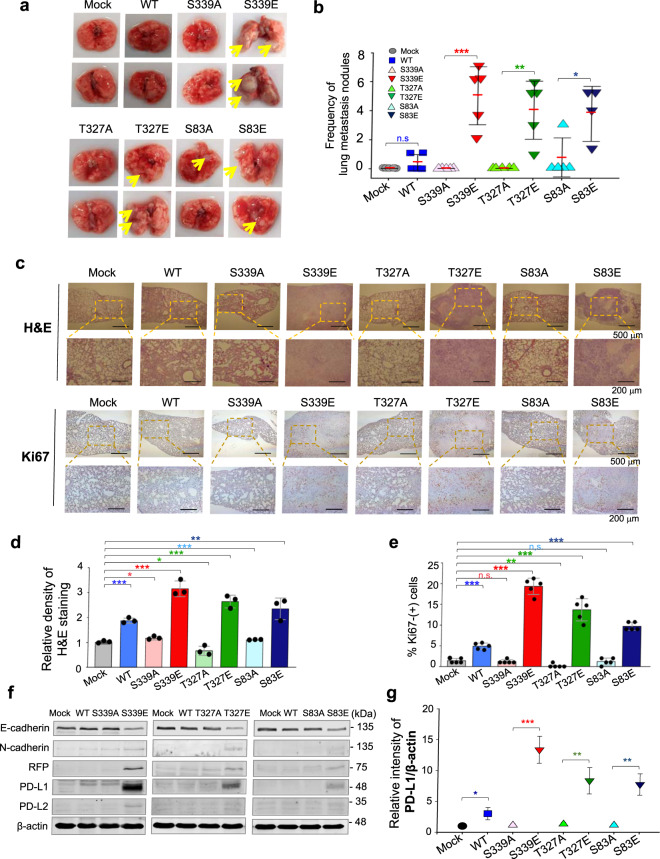
Phosphorylation of vimentin at S339, T327, and S83 accelerates tumorigenesis by increasing the expression of PD-L1 in metastatic cancer. NCI-H460 cells expressing RFP-tagged empty vector (Mock), wild-type (WT) vimentin and S339A, S339E, T327A, T327E, S83A, and S83E mutants were injected intravenously into the tail-veins of four-week-old BALB/c nude mice, and the tumorigenic and metastatic properties were evaluated after 8 weeks. **a** Representative lung tumors from the mouse model. **b** The number of metastatic lung tumors was counted and plotted. (*n* = 5). Data are presented as mean ± SD. **c** Representative H&E staining (upper panel) and Ki-67 staining (lower panel) were performed using lung tissue from the mice. **d** The relative density of H&E staining was analysed and plotted. *p < 0.05; **p < 0.01; ***p < 0.001; (*n* = 3). Data are presented as mean ± SD of three independent experiments (significantly different as compared with experimental control). **e** The populations of Ki-67 positive cells were analysed and plotted (*n* > at least 400 cells in each experiment). *p < 0.05; **p < 0.01; ***p < 0.001; (*n* = 5). Data are presented as mean ± SD of three independent experiments (significantly different as compared with experimental control). **f** Immunoblotting was performed using lung tissue lysates from each mouse model. E-Cadherin, N-cadherin, RFP, PD-L1, PD-L2, and β-actin were detected using specific antibodies. **g** The relative intensity value of PD-L1 was normalized to that of β-actin and plotted. *p < 0.05; **p < 0.01; ***p < 0.001; (*n* = 3). All experiments were performed at least three independent ex*p*eriments. n.s. no significant.

To evaluate whether p-vimentin induces metastasis in an in vivo model, cells expressing various versions of vimentin were injected into the tail veins of BALB/c nude mice (*n* = 5). In mice injected with cells expressing phosphomimetics, the frequency of lung metastatic nodules was higher than in mice that received mock, wild-type, or non-phosphomimetic cells (Fig. 4a, b). In addition, survival times of mice injected with cells expressing S339E or S83E were shorter than those of mice that received non-phosphomimetics (Supplementary Fig. 4). H&E and Ki67 staining revealed upregulated cell proliferation by expression of S339E, T327E, and S83E based on intensity (Fig. 4c–e), indicating that phosphorylation of vimentin at S339, T327, and S83 increases metastasis and tumorigenicity in vivo.

The next question was how p-vimentin induces metastasis and tumorigenicity. Because PD-L1 expression is a clinicopathological feature of LUAD [36], expression of PD-L1 and PD-L2 was analysed using tissue extracts (Fig. 4f, g). Notably, tissues expressing S339E showed the highest level of PD-L1, the highest frequency of metastatic nodules and tumorigenic proliferation, and the shortest OS (Fig. 4f, g). PD-L2 upregulation in tissues expressing phosphomimetic mutants was weak. Among various cells, *CD274* (encoding PD-L1) level was highest in those expressing S339E (Supplementary Fig. 3).

### Suppression of pro-metastatic activity by vimentin blocking is rescued by vimentin phosphorylation at S339

**Fig. 5:**
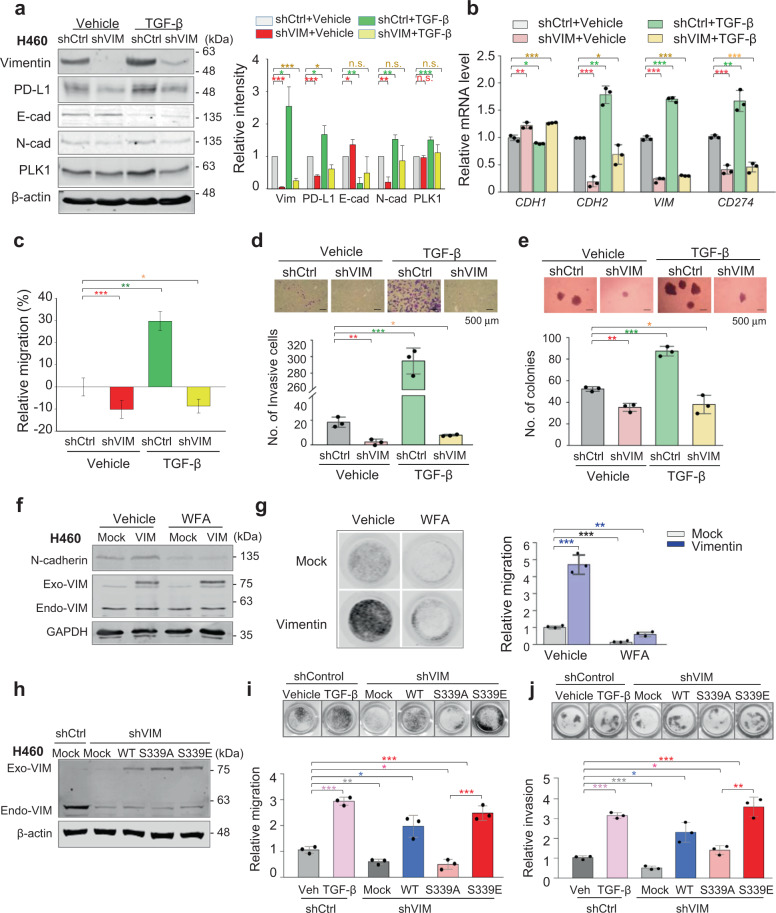
Suppression of pro-metastatic activity in cells by blocking vimentin is rescued by the expression of vimentin phosphomimetic at S339. NCI-H460 cells were infected by lentiviral vimentin shRNA and then treated with TGF-β for 48 hours (**a**–**c**). **a** Immunoblot analyses were performed using anti-vimentin, anti-PD-L1, anti-N-cadherin, anti-E-cadherin, anti-PLK1, and anti-β−actin (left panel). The band intensity values were quantified using LI-COR Odyssey software (Li-COR Biosciences), normalized, and plotted (right panel). *p < 0.05; **p < 0.01; ***p < 0.001; (*n* = 3). n.s., not significant. **b** QRT-PCR was performed for *CDH1, CDH2*, *VIM*, and *CD274* in NCI-H460 cells with depleted vimentin. *p < 0.05; **p < 0.01; ***p < 0.001; (*n* = 3). Data are presented as mean ± SD. **c** NCI-H460 cells depleted of vimentin and treated with TGF-β were subjected to a wound healing assay, as shown in Supplementary Figure 5d. The scratch recovery efficiency after 72 hours was analysed using NIS-Elements Imaging software (Nikon, Japan), and the relative migration distance compared with the control was plotted. *p < 0.05; **p < 0.01; ***p < 0.001; (*n* = 3). Data are presented as mean ± SD. **d** NCI-H460 cells depleted of vimentin were treated with TGF-β. The cells that invaded the bottom layer surface were stained with 0.05% crystal violet dye, and the stained cells were counted. *p < 0.05; **p < 0.01; ***p < 0.001; (*n* = 3). Data are presented as mean ± SD. **e** A colony formation assay was performed with NCI-H460 cells depleted of vimentin, as described in the Materials and Methods. After 2 weeks, the colonies formed in agar were counted after 0.005% crystal violet staining. *p < 0.05; **p < 0.01; ***p < 0.001; (*n* = 3). Data are presented as mean ± SD. **f** NCI-H460 cells expressing RFP-tagged empty vector (Mock) and vimentin (VIM) were treated with the vimentin inhibitor withaferin-A for 48 hours. Immunoblot analyses were performed using anti-vimentin, anti-N-cadherin, and anti-GAPDH. Endo-VIM, endogenous vimentin; Exo-VIM, exogenous vimentin. **g** NCI-H460 cells expressing vimentin and treated with withaferin-A for 3 days were subjected to a Transwell migration assay. Three days after seeding, the cells on the bottom layer surface were stained with 0.05% crystal violet dye, and the intensity values were measured using an Odyssey infrared imaging system (LI-COR Biosciences) and plotted (right panel). *p < 0.05; **p < 0.01; ***p < 0.001; (*n* = 3). Data are presented as mean ± SD. Mouse vimentin mutants were expressed in NCI-H460 cells after depleting endogenous human vimentin using shRNA (**h** and **i**). **h** Immunoblot analyses were performed using anti-vimentin, anti-RFP, and anti-β−actin. Endo-VIM, endogenous vimentin; Exo-VIM, exogenous vimentin. **i** Cells expressing wild-type or mutant vimentin were subjected to a Transwell migration assay. As a positive control of migration, cells were treated with TGF-β. Images of the Transwell cell migration assay were collected, and the intensity values of the stained cells were measured using an Odyssey infrared imaging system (LI-COR Biosciences) and plotted. *p < 0.05; **p < 0.01; ***p < 0.001; (*n* = 3). Data are presented as mean ± SD. **j** An invasion assay was performed using NCI-H460 cells expressing murine vimentin after the depletion of human vimentin. Five days after seeding, the cells that had invaded the bottom layer surface were stained with 0.05% crystal violet dye, and the relative absorbance was plotted. *p < 0.05; **p < 0.01; ***p < 0.001; (*n* = 3). Data are presented as mean ± SD of three independent experiments (significantly different as compared with experimental control).

We next focused on the relationship between vimentin and PD-L1. Vimentin depletion using shRNA during TGF-β-induced EMT downregulated N-cadherin and PD-L1 compared with the control (Fig. 5a; Supplementary Fig. 5a). TGF-β upregulated PD-L1, which was suppressed by vimentin depletion (Fig. 5a). The patterns of PD-L1 protein and mRNA were similar to those of vimentin in both NCI-H460 and A549 cells (Fig. 5a, b; Supplementary Fig. 5b, c), suggesting importance of vimentin expression in the expression of PD-L1 in LUAD. PLK1 level was weakly downregulated by vimentin depletion compared with the control, indicating the effect of vimentin-activated EMT signaling on PLK1 expression (Fig. 5a).

The effects of vimentin deficiency on cell migration and invasiveness in TGF-β-induced EMT were observed (Fig. 5c, d; Supplementary Fig. 5d). The relative motility of vimentin-depleted cells was reduced by ≈10% at 72 hours compared to control (Fig. 5c). TGF-β-induced migration was decreased by vimentin depletion. In Transwell-based invasion assay, vimentin depletion reduced the number of invading cells induced by TGF-β (Fig. 5d). In colony formation assay, ≈52 control-cell colonies formed versus fewer than 35 colonies of vimentin-depleted cells when NCI-H460 cells were cultured with TGF-β in agar (Fig. 5e), indicating the importance of vimentin for LUAD tumorigenesis.

We also analysed cell migration in the presence of the vimentin inhibitor withaferin-A [37] (Fig. 5f, g). Withaferin-A treatment reduced N-cadherin expression in cells expressing vimentin (Fig. 5f). Motility of cells expressing vimentin was ≈4.7-fold higher than that of the mock. This motility decreased dramatically by 8.2-fold with withaferin-A treatment compared with vehicle cells expressing vimentin (Fig. 5g, right panel). This indicates that vimentin inhibition by withaferin-A reduces cell migration activity. To confirm the pro-metastatic property of phosphorylated vimentin, murine vimentin was expressed in human vimentin–depleted NCI-H460 cells (Fig. 5h–j). Migration activity and invasiveness recovered with S339E expression in vimentin-depleted cells (Fig. 5i, j). Taken together, our results indicate vimentin function is critical for inducing cell migration, invasiveness, PD-L1 expression, and tumorigenesis in LUAD cells.

### Phosphorylation of vimentin upregulates expression of PD-L1 through activation of Stat3 and TGF-β signaling

**Fig. 6:**
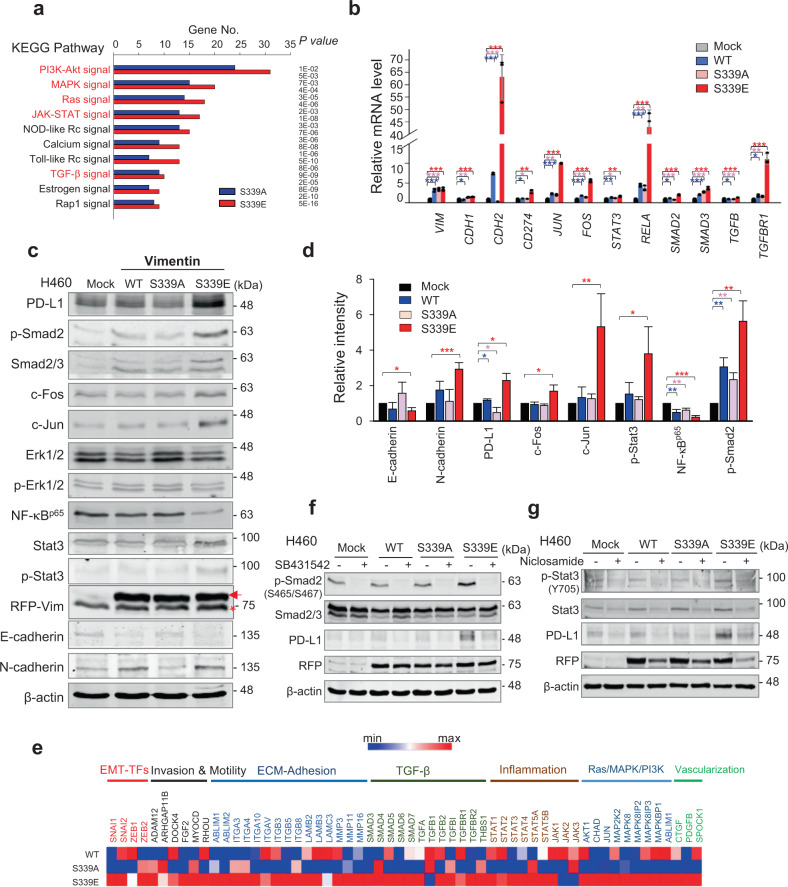
Phosphorylation of vimentin upregulates the expression of PD-L1 through the activation of Stat3 and Smad2 signaling. **a** The transcriptome data were clustered by gene probes with fold changes >1.5, which revealed that the levels of 1503 genes were changed significantly in NCI-H460 cells expressing vimentin. KEGG pathways were analysed, and the signaling pathways with higher gene numbers in cells expressing S339E than in those expressing S339A are displayed. **b** QRT-PCR was performed for *CDH1, CDH2*, *CD274, VIM, JUN, FOS, STAT3, RELA, SMAD2, SMAD3, TGFB*, and *TGFBR1* in NCI-H460 cells expressing vimentin. *p < 0.05; **p < 0.01; ***p < 0.001; (*n* = 3). Data are presented as mean ± SD of three independent experiments (significantly different as compared with experimental control). **c** Immunoblot analyses were performed using specific antibodies. Arrow, exogenous vimentin band; *, non-specific band. **d** The band intensity values were quantified using LI-COR Odyssey software (Li-COR Biosciences), normalized, and plotted (right panel). *p < 0.05; **p < 0.01; ***p < 0.001; (*n* = 3). **e** Transcriptome comparison among the gene profiles of cells expressing wild-type, S339A, and S339E vimentin. The MORPHEUS program was used to visualize the expression levels of genes related to TGF-β signaling, Ras/MAPK/PI3K signaling, transcriptional factors of the EMT, ECM-adhesion, invasion, motility, vascularization, colonization, and niche. **f** NCI-H460 cells expressing vimentin were treated with SB431542, an inhibitor of Smad2/3 phosphorylation, for 48 hours. Immunoblot analyses were performed using anti-RFP, anti-PD-L1, anti-Smad2/3, anti-p-Smad2^S465/S467^, and anti-β−actin. **g** NCI-H460 cells expressing vimentin were treated with niclosamide, a Stat3 inhibitor, for 48 hours and then subjected to immunoblotting. Immunoblot analyses were performed using anti-RFP, anti-PD-L1, anti-Stat3, anti-p-Stat3^Y705^, and anti-β−actin. All experiments were performed at least three independent experiments.

We performed microarray analysis for transcriptome profiling to identify the signaling factors regulating PD-L1 expression in the presence of vimentin. Transcriptome data were clustered by gene probes with fold changes >1.5, revealing that levels of 1,503 genes changed significantly in cells expressing vimentin. To understand the signaling associated with vimentin phosphorylation at S339, signaling pathways were analysed using KEGG pathways (Fig. 6a). Expression of S339E affected PI3K/Akt, MAPK, Ras, JAK-STAT, and TGF-β signaling. Because the downstream factors of those signaling pathways (NF-κB [38], AP-1 [39], Stat3 [40, 41], and Smad2/3 [42, 43]) are possible transcriptional factors for PD-L1 expression, we used qRT-PCR to observe them in cells expressing wild-type or mutant vimentin (Fig. 6b). *CDH2* and *CD274* levels were higher in cells expressing S339E than S339A when *VIM* levels were similar. Likewise, mRNA levels of *JUN*, *STAT3*, and *TGFB* were higher in cells expressing S339E than in those expressing S339A. Protein levels of c-Jun, Stat3, and Samd2/3 were similar to those of mRNA (Fig. 6c, d). In addition, phosphorylation levels of Smad2 and Stat3 (Fig. 6c, d) and mRNA levels of *TGFB* and *STAT3* increased in cells expressing S339E, suggesting TGF-β/Smad and STAT signaling to be involved in expression of PD-L1. TGF-β treatment also upregulated level of p-Smad2 increased by S339E expression (Supplementary Fig. 6a). Moreover, gene changes and KEGG pathway analysis from microarray were compared between cells expressing S339E and S339A (Fig. 6e; Supplementary Fig. 6b), showing that phosphomimetics upregulated expression of genes related to TGF-β signaling, Ras/MAPK signaling, invasion, and metastasis.

To determine which signaling pathway was the most important to PD-L1 expression in cells expressing S339E, specific inhibitors SB431542, niclosamide, and U0126, were used to block signals mediated by TGF-β/Smad, STAT, and MAPK, respectively (Fig. 6f, g; Supplementary Fig. 6c). Treatment with SB431542 reduced levels of p-Smad2 and PD-L1 in cells expressing S339E (Fig. 6f), indicating that TGF-β/Smad signaling regulates PD-L1 expression. Niclosamide treatment to inhibit Stat3 reduced PD-L1 levels, indicating that STAT signaling is involved in PD-L1 expression (Fig. 6g). Specific ERK inhibitor U0126 did not affect PD-L1 level (Supplementary Fig. 6c). Therefore, TGF-β/Smad signaling and STAT signaling are possible triggers for PD-L1 expression in cells containing p-vimentin^S339^.

### p-Vimentin^S339^ recruits p-Smad2/3 to cell nuclei for PD-L1 expression, increasing cell viability in the presence of cytotoxic Jurkat T cells

**Fig. 7:**
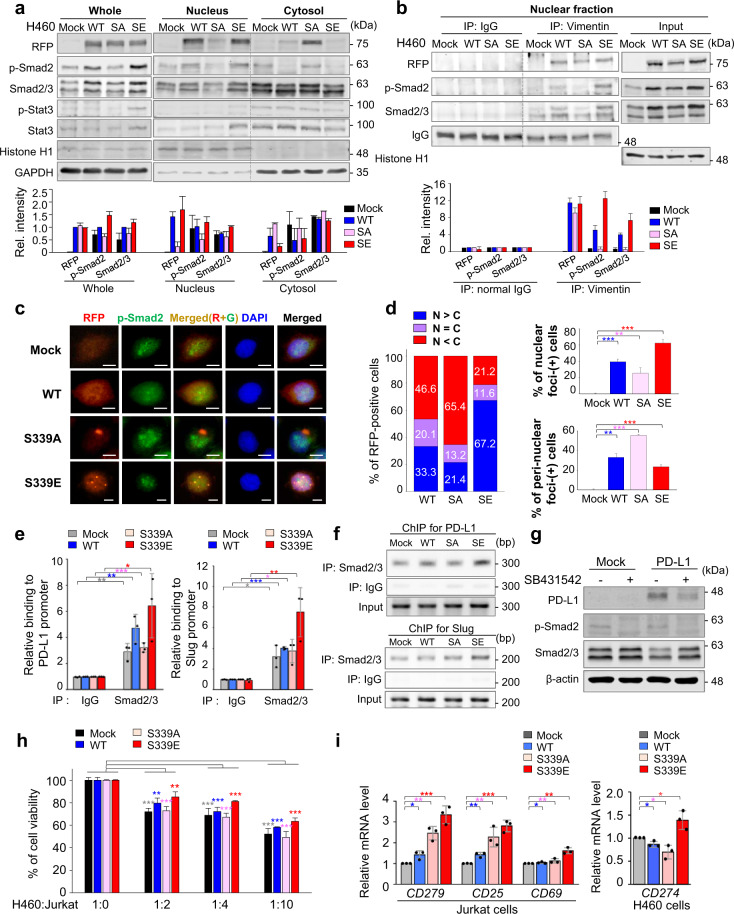
p-Smad2/3 is recruited to the promoter of PD-L1 by phosphomimetic vimentin and upregulates PD-L1 expression, increasing cell viability in the presence of cytotoxic Jurkat T cells. **a** Immunoblotting was performed using whole, nuclear, and cytoplasmic lysates of NCI-H460 cells. Anti-RFP, anti-p-Smad2, anti-Smad2/3, anti-p-Stat3, and anti-Stat3 antibodies were used. Histone H1, Nuclear loading marker; GAPDH, Cytoplasmic loading control. **b** Immunoprecipitation was done using nuclear lysates of NCI-H460 cells. Anti-normal IgG and anti-vimentin were used for the immunoprecipitation, and anti-RFP, anti-p-Smad2, anti-Smad2/3, and anti-histone H1 antibodies were used for immunoblotting. **c** NCI-H460 cells were stained with anti-RFP (Sigma; red) and anti-p-Smad2 (Cell Signaling; green). Nuclear DNA was stained by DAPI. **d** Quantification of the population of cells in the cytoplasm, nucleus, and both. *n* > 1000. Scale bar, 5 μm. **e** ChIP assays for Smad2/3 binding to the PD-L1 promoter. Assays were performed on chromatin fragments using antibody to Smad2/3 and normalized to pre-immune normal IgG. Immunoprecipitated fractions were assayed by real time-PCR for binding to the PD-L1 promoters. Data are presented as mean ± SD of three independent experiments (significantly different as compared with experimental control). *p < 0.05; **p < 0.01; ***p < 0.001; (*n* = 3). **f** ChIP assays for Smad2/3 binding to the PD-L1 promoter. Assays were performed on chromatin fragments using antibody to Smad2/3 and normalized to pre-immune normal IgG. Immunoprecipitated fractions were assayed by PCR for binding to the PD-L1 promoters. The PCR products were visualized in agarose gel. **g** Inhibition of Smad2/3 using specific inhibitor SB431542 reduced the expression of PD-L1 in A549 cells transiently expressing PD-L1. Immunoblotting was performed using anti-PD-L1, anti-p-Smad2, anti-Smad2/3, and anti-β-actin antibodies. **h** The viability of NCI-H460 cells expressing vimentin was measured when cells were co-cultured with cytotoxic T cells. The ratio between NCI-H460 cells and Jurkat T cells was 1:0, 1:2, 1:4, and 1:10, as indicated. Data are presented as mean ± SD of three independent experiments (significantly different as compared with experimental control). **p < 0.01; ***p < 0.001; (*n* = 3). **i** Relative mRNA levels for CD69, CD25, and CD27 were measured by real-time PCR in Jurkat cells. The relative mRNA level of CD274 was measured in NCI-H460 cells expressing vimentin. Data are presented as mean ± SD of three independent experiments (significantly different as compared with experimental control). *p < 0.05; **p < 0.01; ***p < 0.001; (*n* = 3). All experiments were performed at least three independent experiments.

We further investigated how p-vimentin regulates Smad2/3 and Stat3 in PD-L1 expression. Based on the functional rationale in mast cells [44], we hypothesized that p-vimentin could regulate translocation of p-Smad2/3 or p-Stat3 into the nucleus for PD-L1 expression. Locations of p-Smad2/3 and p-Stat3 were observed using fractionation assays (Fig. 7a). Notably, S339E vimentin mainly located to the nucleus, unlike non-phosphomimetics. Nuclear p-Smad2/3 was higher in cells expressing S339E than S339A, but p-Stat3 was not. To understand whether p-vimentin interacts with p-Smad2/3 or p-Stat3, immunoprecipitation assay was performed using whole-cell lysates (Supplementary Fig. 7a) and nuclear fractions (Fig. 7b). Results indicate S339E vimentin interacted with Smad2/3 and p-Smad2 but not p-Stat3. Thus, p-vimentin^S339^ interacts with p-Smad2 within the Smad2/3 complex for translocation into the nucleus. Immunostaining revealed phosphomimetics at S339 localized in the nucleus, colocalizing with p-Smad2, whereas non-phosphomimetics were not (Fig. 7c, d; Supplementary Fig. 7b). To determine whether p-Smad2 triggers expression of PD-L1, chromatin immunoprecipitation (ChIP) was performed using anti-Smad2/3 and anti-Stat3 (Fig. 7e, f; Supplementary Fig. 7c-e). Nuclear Smad2/3 was detected in the promoter region of PD-L1 in cells expressing S339E, but Stat3 was not found on ChIP assay. To confirm whether Smad2/3 regulates PD-L1 expression, SB431542 was administered to A549 cells expressing PD-L1 (Fig. 7g) and markedly reduced expression of PD-L1 by inhibiting Smad2/3 activation. These observations provide a strong rationale for translocation of a p-vimentin/p-Smad2/3 complex into the nucleus for the transcriptional activation of PD-L1.

To demonstrate whether PD-L1 expression in cells expressing S339E caused immunosuppression, cell viability was analysed in cocultured with cytotoxic Jurkat T cells (Fig. 7h, i). Cells expressing S339E had the highest survival rate of all the cells tested (Fig. 7h) because *CD274* (PD-L1 mRNA) level was highest (Fig. 7i). In Jurkat cells cocultured with NCI-H460 cells expressing S339E at a ratio of 2:1, mRNA levels of the T cell activators *CD69* and *CD25* and the level of *CD279* (encoding PD-1, a receptor of PD-L1) were also higher than in other cells (Fig. 7i). Thus, in Jurkat cells cocultured with NCI-H460 cells expressing S339E, *CD69* and *CD25* were activated for immune surveillance. However, high expression of *CD274* overcomes the attack of cytotoxic T cells, enabling immune escape for cells expressing S339E vimentin.

**Fig. 8:**
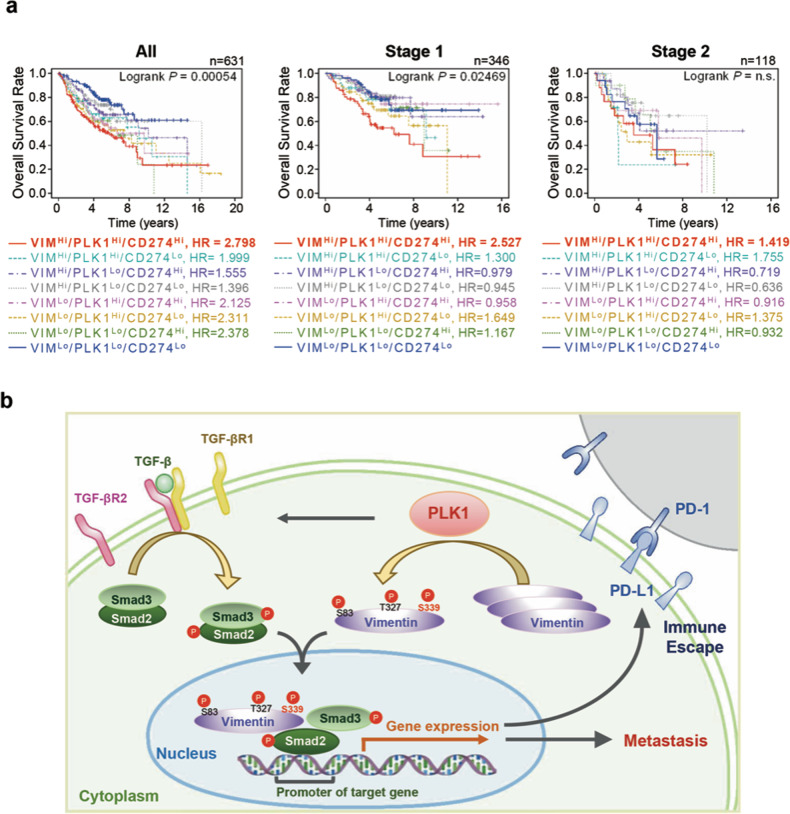
Clinical implication and plausible action mechanism of vimentin in promoting metastasis and immune suppression. **a** Clinical association between the expression of *PLK1/VIM/CD274* and the cumulative OS times of LUAD patients. The survival times of all (*n* = 631), stage 1 (*n* = 346), and stage 2 (*n* = 118) LUAD patients were analysed according to their *PLK1/VIM/CD274* expression levels using KM PLOTTER. High (Hi) *vs*. low (Lo) expression was split by mean value. n.s., not significant. **b** Phosphorylation of vimentin by PLK1 at S339, T327, and S83 facilitates metastatic tumorigenesis and immune escape through the activation of TGF-β/Smad signaling and the expression of PD-L1 in LUAD. p-Vimentin ^S339^ upregulates PD-L1 by activating Smad2/3 and interacting with p-Smad2 for nuclear translocation, where it recruits them to the PD-L1 promoter regions for transcriptional activation, immune escape, and tumor survival. Vimentin acts as a coordinator for metastatic tumorigenesis in LUAD via its phosphorylation, activation of TGF-β signaling for metastasis, and interaction with p-Smad2/3, which it recruits to the PD-L1 promoter for tumor survival.

The clinical relationship between the expression of *PLK1/VIM/CD274* and cumulative OS time was analysed in LUAD patients (Fig. 8a, Supplementary Table 6). OS times of LUAD patients with high expression of *PLK1/VIM/CD274* were significantly lower than those of patients with low expression of *PLK1/VIM/CD274* (*n* = 631, HR = 2.798, log rank *P* = 0.0005) (Fig. 8a, left panel). In clinical analysis of adenocarcinoma patients with stage 1 (Fig. 8a, middle), patients with high *PLK1/VIM/CD274* had shorter OS times than those with low *PLK1/VIM/CD274* (*n* = 346, HR = 2.527, log rank *P* = 0.0246). Likewise, metastatic stage 2 LUAD patients with high *PLK1/VIM/CD274* had shorter OS times than those with low *PLK1/VIM/CD274* expression (*n* = 118, HR =1.419, log rank *P* = 0.6069) (Fig. 8a, right). Therefore, high expression of vimentin, PLK1, and PD-L1 could be used as a negative index for survival in primary and metastatic LUAD patients.

## Discussion

Correlation between expression of vimentin, a canonical marker of EMT, and malignancy has been broadly studied; [14–17, 21] however, how vimentin regulates tumor metastasis and survival remains under investigation. Here, we report phosphorylation of vimentin by PLK1 at S339, T327, and S83 to facilitate metastatic tumorigenesis and immune escape by activating TGF-β/Smad signaling and inducing expression of PD-L1 in LUAD. As the underlying mechanism of vimentin activation of metastasis and PD-L1 expression, vimentin phosphorylated by PLK1 interacts with p-Smad2/3 for nuclear translocation (Fig. 8b), and nuclear p-Smad2/3 binds to the PD-L1 promoter to trigger PD-L1 expression, enabling immune escape and tumor survival, as analysed by coculture with cytotoxic T cells. Phosphorylated vimentin functions as a shuttle protein, interacting with p-Smad2/3 for translocation to the nucleus. The evidence indicates that vimentin phosphomimetic at S339 in the nucleus forms a complex with p-Smad2/3, whereas non-phosphomimetic vimentin is mainly located in the cytoplasm and does not interact with p-Smad2/3 (Fig. 7). The increased PLK1 in TGF-β−induced EMT was downregulated by vimentin depletion (Fig. 5a), indicating the effects of vimentin-mediated EMT on PLK1 expression. Thus, PLK1-induced phosphorylation of vimentin is a coordinator of metastatic tumorigenesis in LUAD through activation of TGF-β signaling for metastasis and expression of PD-L1 by p-Smad2/3 for tumor survival.

The correlation between *VIM* expression and patient survival time was significant in LUAD, but not in LUSQ (Supplementary Fig. 1). *VIM* overexpression is an independent prognostic indicator of poor survival in NSCLC patients [15, 45]. Our more detailed categorization of NSCLC revealed *VIM* overexpression correlates with shorter survival time only in LUAD patients. However, PLK1 is highly upregulated in both LUSQ and LUAD patients and is a promising target for NSCLC treatment [46]. In our clinical analysis of primary and metastatic adenocarcinoma patients with stage 1 or 2 tumors, those with high *PLK1/VIM* expression had shorter OS times than those with low *PLK1/VIM* expression (Fig. 1). Thus, simultaneously high levels of both PLK1 and vimentin could be used as a negative index for survival in primary and metastatic LUAD patients.

Phosphorylation of vimentin has been studied in mitotic dynamics [34, 35], smooth muscle contractions [47, 48], and cancer metastasis [23, 24, 49], revealing that phosphorylation is a main regulatory mechanism of its function through disassembly of IFs. Here, we reported several sites where PLK1 phosphorylates vimentin as metastatic regulatory residues, including S339, T327, and S83. Among them, phosphorylation at S339 and T327 is effective for metastasis and immune escape, as shown by nodule sizes and expression of PD-L1 in an in vivo model. The S339 and T327 residues are located in the coil 2 region of the rod domain of vimentin, which is structurally composed of a head, an alpha helix, and a tail domain [50]. The C328 residue of vimentin is a crucial site for optimal organization and remodeling in cells through disulfide bond formation [51]. Because T327 residue is next to C328 residue, a critical amino acid for vimentin organization, phosphorylation of T327 could affect vimentin IF reorganization. According to a structure-based protein stability prediction based on single-point mutations [52], the energy change between folded and unfolded status, ▵▵G (free energy gap difference between wild-type and mutant proteins), is negative in mutants at C328 and non-phosphomimetics at S327 and S339, indicating those mutants are less stable than wild-type (Supplementary Fig. 8). However, the ▵▵G of phosphomimetic at S327 or S339 is positive, indicating mutants phosphorylated at S327 and S339 are more stable than wild-type, suggesting their physiological importance to IF reorganization. In previous studies [22, 23, 49], assembly and disassembly of vimentin IFs were regulated by phosphorylation status. The cells expressing S339E forming IFs represented ≈15% of all cells, while 53% of cells expressing S339A formed IFs bundles (Supplementary Fig. 9), indicating that phosphorylation of vimentin leads to disassembly of IFs to increase motility of cancer cells through lamellipodia formation [49]. In addition, phosphomimetics are located at nuclear foci, whereas non-phosphomimetics locate at peri-nuclear foci as a juxta nuclear knot for adhesion [53], indicating phosphorylation of vimentin by PLK1 increases nuclear translocation to facilitate translocation of p-Smad2/3 for expression of PD-L1 and mesenchymal genes. Thus, phosphorylation of vimentin directly increases cell motility through IFs dynamics and indirectly upregulates gene expression by functioning as a shuttle protein for p-Smad2/3 translocation into the nuclei.

Upregulation of PD-L1 in cells having S339E increased cancer viability by defending the cells from the immune response of activated T lymphocytes, as determined by *CD69* and *CD25* expression. PD-1 and its ligand PD-L1 are the most promising targets found in recent immune-oncotherapy research. PD-1 is an immunosuppressive receptor expressed on T cells that manages tumor immune escape [54]. On the other hand, PD-L1 expressed in solid tumors including melanoma and carcinomas of the lung, breast, bladder, kidney, pancreas, oesophagus, and ovary is an important prognostic marker for immunotherapies that use anti-PD-L1 [54–56]. Interaction between PD-1 and PD-L1 inhibits T lymphocyte activation, induces apoptosis of tumor-specific T cells, and promotes differentiation of T cells, which eventually leads to immune evasion of tumor cells [54]. Intriguingly, in our in vivo metastatic model, PD-L1 was highly upregulated when phosphomimetics were expressed. In particular, vimentin with a phosphomimetic at S339 strongly triggered PD-L1 expression, suggesting PLK1-mediated phosphorylation of vimentin enabled escape from the immune response through PD-L1 expression, which promotes metastasis and tumorigenicity in vivo. Current immunotherapies show fast progression and relatively weak adverse effects when patients respond to treatment with anti-PD-1 or anti-PD-L1 drugs such as nivolumab and atezolizumab [57, 58]. However, the response rates to those immunotherapies are low, ≈20% (e.g., nivolumab, 18.8% NSCLC, PD-L1 + ) [59–61]. Increasing PD-L1 expression could improve their therapeutic effects. Because p-vimentin increased PD-L1 level, high levels of PLK1 and vimentin could increase the response rate of LUAD patients treated with anti-PD-1 or anti-PD-L1 therapeutics.

In this study, vimentin phosphorylated by PLK1 triggers TGF-β/Smad2 signaling and translocates to the nucleus with p-Smad2/3 to promote PD-L1 expression, which leads to metastasis and immunosuppression for tumorigenesis in LUAD. Simultaneously high levels of PLK1 and vimentin could be used as a marker to increase immunotherapeutic response rates among LUAD patients.

## Supplementary information

Supplementary Information

Supplementary Tables

Supplementary Figure 1.

Supplementary Figure 2.

Supplementary Figure 3.

Supplementary Figure 4.

Supplementary Figure 5.

Supplementary Figure 6.

Supplementary Figure 7.

Supplementary Figure 8.

Supplementary Figure 9.

Supplementary Figure 10.

Dataset 1
