# Correction: PLK1/vimentin signaling facilitates immune escape by recruiting Smad2/3 to PD-L1 promoter in metastatic lung adenocarcinoma

**DOI:** 10.1038/s41418-021-00842-8

**Published:** 2021-08-17

**Authors:** Hay-Ran Jang, Sol-Bi Shin, Chang-Hyeon Kim, Jae-Yeon Won, Rong Xu, Da-Eun Kim, Hyungshin Yim

**Affiliations:** 1 Department of Pharmacy, College of Pharmacy, Hanyang University, Ansan, Gyeonggi-do Korea; 2 Institute of Pharmaceutical Science and Technology, Hanyang University, Ansan, Gyeonggi-do Korea

**Keywords:** Cancer microenvironment, Prognostic markers

Correction to: *Cell Death & Differentiation* 10.1038/s41418-021-00781-4, published online 7 May 2021

The original version of this article unfortunately contained a mistake. After the publication of the article, the author found labelling error in Figs. 1d and 8a. The correct labelling is Stage 1 and Stage 2 instead of Stage N1 and Stage N2. Due to these errors, the text, the figures and the figure legends need to be corrected. The authors apologize for the mistake. The original article has been corrected.

