# Supplementary material for: PLK1/vimentin signaling facilitates immune escape by recruiting Smad2/3 to PD-L1 promoter in metastatic lung adenocarcinoma
Source: Cell Death Differ. 2021 May 7;28(9):2745–64. doi: 10.1038/s41418-021-00781-4 (PMC8408167; doi:10.1038/s41418-021-00781-4)
Supplement: Supplementary file 1 — Supplementary Information [file 41418_2021_781_MOESM1_ESM.docx]

**Supplementary Information**

Hay-Ran Jang^1^, Sol-bi Shin^1,2^, Chang-Hyeon Kim^1^, Jae-Yeon Won^1^, Rong Xu^1^, Da-Eun Kim^1^, and Hyungshin Yim^1,2, *^

^1^Department of Pharmacy, College of Pharmacy**,** Hanyang University, Ansan, Gyeonggi-do 15588, Korea

^2^**Institute of Pharmaceutical Science and Technology,** Hanyang University, Ansan, Gyeonggi-do 15588, Korea

^*^Corresponding author

Hyungshin YIM

Address: Department of Pharmacy, College of Pharmacy, **Institute of Pharmaceutical Science and Technology,** Hanyang University, Ansan, Gyeonggi-do 15588, Korea

Phone: +82-31-400-5810

FAX: +82-31-400-5958

E-mail: [hsyim@hanyang.ac.kr](mailto:hsyim@hanyang.ac.kr)

The authors declare no potential conflicts of interest.

Keywords: PLK1, vimentin, PD-L1, metastasis, immunosuppression

Running Title: p-Vimentin regulates immune escape in lung adenocarcinoma

**SUPPLEMENTARY Figure legends**

**Supplementary Figure 1.** **Clinical relevance of *PLK1* or *VIM* expression and the survival times of patients with lung cancer, lung adenocarcinoma, or squamous carcinoma.** **a** The OS times of patients with lung cancer (left panel, *n*=1926), squamous carcinoma (middle panel, *n*=524), and adenocarcinoma (right panel, *n*=720) were analysed according to their *PLK1* expression levels using KM PLOTTER. **b** The OS times of patients with lung cancer (left panel, *n*=1926), squamous carcinoma (middle panel, *n*=524), and adenocarcinoma (right panel, *n*=720) were analysed according to their *VIM* expression levels using KM PLOTTER.

**Supplementary Figure 2. Vimentin is phosphorylated by PLK1 at T327, S339, S459, and S83 residues. a** NCI-H460 were treated with 2.5 ng/ml of TGF-β for 48 hours to induce EMT. Immunoprecipitation of cell lysates was performed with anti-PLK1 antibody or normal IgG followed by immunoblotting with anti-vimentin antibody. **b** A purified GST-tagged active form of PLK (TD) from baculovirus-infected cells was used in a kinase assay with radioactive ATP and purified GST-vimentin (as the substrate). GST-TCTP (TCTP) was used as the positive control. **c** NCI-H460 cells were treated with 2 mM hydroxyurea and 100 ng/ml nocodazole for 12 hours. 5 ng/ml TGF-β was treated for 48 hours. Immunoblotting was performed to measure the phosphorylation of vimentin at S83 using anti-p-S83-vimentin and anti-vimentin antibodies in NCI-H460 cells. **d** The phosphorylation residues of vimentin are conserved in several species, including *Homo sapiens, Pan troglodytes, Canis lupus familiaris, Rattus norvegicus,* and *Mus musculus.*

**Supplementary Figure 3.** **Relative mRNA levels in NCI-H460 cells expressing various versions of vimentin.** RFP-tagged wild-type (WT) vimentin and S339A, S339E, T327A, T327E, S83A, and S83E mutants were expressed in NCI-H460 cells. Cells were treated with doxycycline for 48 hours to express the RFP-tagged vimentin. QRT-PCR was performed for *CDH1,* *CDH2, SNAI1, SNAI2, CD274*, and *VIM*, and the relative mRNA levels were plotted.

**Supplementary Figure 4. Phosphorylation of vimentin at S339, T327, and S83 reduces the survival times of mice in metastatic cancer.** NCI-H460 cells expressing RFP-tagged wild-type (WT) vimentin and S339A, S339E, T327A, T327E, S83A, and S83E mutants were injected intravenously into the tail-veins of four-week-old BALB/c nude mice, and the metastatic properties and survival times were evaluated after 8 weeks (*n* =5). Survival times of mice with various versions of vimentin were determined and plotted.

**Supplementary Figure 5.** **Depletion of vimentin using shRNA reduced pro-metastatic activities in cells.** A549 cells were infected by lentiviral vimentin shRNA and then treated with TGF-β for 48 hours (a-c). **a** Scheme of experiments. A549 cells were infected with lentiviral vimentin shRNA and then treated with TGF-β for 48 hours. **b** Immunoblot analyses were performed using anti-vimentin, anti-PD-L1, anti-N-cadherin, anti-E-cadherin, anti-PLK1, and anti-β−actin (left panel). The band intensity values were quantified using LI-COR Odyssey software (Li-COR Biosciences), normalized, and plotted (right panel). **c** QRT-PCR was performed for *CDH1, CDH2*, *VIM*, and *CD274* in NCI-H460 cells with depleted vimentin. **p* <0.05; ***p* <0.01; ****p* <0.001; (*n*=3). Data are presented as mean ± SD. A549 cells were infected by lentiviral vimentin shRNA and then treated with TGF-β for 48 hours. **d** NCI-H460 cells depleted of vimentin and treated with TGF-β were subjected to a wound healing assay. The scratch recovery efficiency after 72 hours was analysed using NIS-Elements Imaging software (Nikon, Japan), and the relative migration distance compared with the control was plotted. **p* <0.05; ***p* <0.01; ****p* <0.001; (*n*=3). Data are presented as mean ± SD.

**Supplementary Figure 6. The signalling pathways in cells expressing S339E. a** RFP-tagged empty vector (Mock), wild-type (WT), S339A, and S339E of vimentin were expressed in NCI-H460 cells and 2.5 ng/ml TGF-β were treated for 48 hours. Immunoblot analyses were performed using specific antibodies (left panel). Arrow, exogenous vimentin band; *, non-specific band. The band intensity values were quantified using LI-COR Odyssey software (Li-COR Biosciences), normalized, and plotted (right panel). ***p* <0.01; ****p* <0.001; (*n*>3). **b** The transcriptome data were clustered by gene probes with fold changes >1.5, which revealed that the levels of 1134 genes and 1137 genes were changed significantly in cells expressing S339A and S339E vimentin, respectively. Using the ClueGO application for Cytoscape, KEGG pathways were analysed. The pathways showing cells expressing S339E but not cells expressing S339A are displayed. **c** NCI-H460 cells expressing vimentin were treated with the Erk1/2 inhibitor U0126 for 48 hours. Immunoblot analyses were performed using anti-RFP, anti-PD-L1, anti-Erk1/2, anti-p-Erk1/2 (T202/Y204), and anti-GAPDH.

**Supplementary Figure 7. Regulation of PD-L1 expression by p-vimentin through localization into the nucleus and activation of the PD-L1 promoter by recruitment of p-Smad2/3. a** NCI-H460 cells were expressed wild-type, S339A, and S339E of vimentin. Immunoprecipitation was done using whole cell lysates. Anti-normal IgG and anti-vimentin were used for the immunoprecipitation, and anti-RFP, anti-p-Smad2, and anti-Smad2/3 antibodies were used for the immunoblotting. Relative intensity was plotted. **b** Cells were stained with anti-RFP (Sigma; red) and anti-vimentin (green). Nuclear DNA was stained by DAPI (blue). The populations of cells in the cytoplasm, nucleus, and both were quantified. n> 1500. Scale bar, 10 μm. **c** Scheme of PD-L1 promoter region. Smad2 binds to the region of CAGA sequences. **d** ChIP assays for Stat3 binding to the PD-L1 promoter. Assays were performed on chromatin fragments using antibody to Stat3 and normalized to pre-immune normal IgG. Immunoprecipitated fractions were assayed by PCR for binding to the PD-L1 promoters. The PCR products were visualized in agarose gel electrophoresis (left panel). **e** Immunoprecipitated fractions were assayed by real time-PCR for binding to the PD-L1 promoters. Data are presented as mean ± SD of three independent experiments (significantly different as compared with experimental control) (right panel).

**Supplementary Figure 8. Analysis of structure-based protein stability predictions based on single point mutations.** The energy change between folded status and unfolded status, 𝚫𝚫G (free energy gap difference between wild-type and mutant proteins), is negative with C328 mutation or non-phosphomimetics at S327 and S339.

**Supplementary Figure 9. Reduction of intermediate filament formation in cells expressing S339E.** NCI-H460 cells expressing wild-type, S339A, and S339E of vimentin were stained with anti-RFP (red) and anti-p-vimentin (green). Nuclear DNA was stained by DAPI. The populations of cells with intermediate filaments formation quantified. n> 500. Scale bar, 10 μm. ****p* <0.001; (*n*>500).

**Supplementary Figure 10. Schemes of experiments.**

**SUPPLEMENTARY Table legends**

**Supplemental Table 1. Sequences of forward (F) and reverse (R) primers used for RT-PCR amplification.**

**Supplemental Table 2. Sequences of forward (F) and reverse (R) primers used for RT-PCR amplification.**

**Supplemental Table 3. Cox regression analysis for the survival of lung adenocarcinoma (LUAD) patients expressing PLK1 and VIM of KM plot used in figure 1c.**

**Supplemental Table 4. Cox regression analysis for the overall survival of LUAD patients expressing PLK1 and VIM of KM plot used in figure 1d.**

**Supplemental Table 5. Types of lung cancer cell lines used in Figure 1e.**

**Supplemental Table 6. Cox regression analysis for the overall survival of LUAD patients expressing PLK1, VIM, and CD274 of KM plot used in figure 8a.**
