# Supplementary material for: PLK1/vimentin signaling facilitates immune escape by recruiting Smad2/3 to PD-L1 promoter in metastatic lung adenocarcinoma
Source: Cell Death Differ. 2021 May 7;28(9):2745–64. doi: 10.1038/s41418-021-00781-4 (PMC8408167; doi:10.1038/s41418-021-00781-4)
Supplement: Supplementary file 2 — Supplementary Tables [file 41418_2021_781_MOESM2_ESM.docx]

| Target Gene | Primer | Sequences |
| --- | --- | --- |
| Human *PLK1* | Forward  Reverse | 5’- AAGAGATCCCGGAGGTCCTA -3’  5’- TCATTCAGGAAAAGGTTGCC -3’ |
| Human *CDH1* | Forward  Reverse | 5’- ACCACCTCCACAGCCACC -3’  5’- GTCCAGTTGGCACTCGCC -3’ |
| Human *CDH2* | Forward  Reverse | 5’- ACAGTGGCCACCTACAAAGG -3’  5’- CCGAGATGGGGTTGATAATG -3’ |
| Human *VIM* | Forward  Reverse | 5’- GAGAACTTTGCCGTTGAAGC -3’  5’- GCTTCCTGTAGGTGGCAATC -3’ |
| Mouse *VIM* | Forward  Reverse | 5’-CGGCTGCGAGAGAAATTGC-3’  5’-CCACTTTCCGTTCAAGGTCAAG-3’ |
| Human *SNAI1* | Forward  Reverse | 5’- GGAAGCCTAACTACAGCGAG -3’  5’- CAGAGTCCCAGATGAGCATTG -3’ |
| Human *SNAI2* | Forward  Reverse | 5’- ACGCCCAGCTACCCAATG -3’  5’- AGGGCGCCCAGGCTCACATA -3’ |
| Human *CD274* | Forward  Reverse | 5′-CAAAGAATTTTGGTTGTGGA-3′  5′-AGCTTCTCCTCTCTCTTGGA-3′ |
| Human *c-Jun* | Forward  Reverse | 5’-ATCCTGAAACAGAGCATGAC -3’  5’-GTTGCTGGACTGGATTATCA-3’ |
| Human *c-fos* | Forward  Reverse | 5’-CTGGCGTTGTGAAGACCAT-3’  5’-TCCCTTCGGATTCTCCTTTT-3’ |
| Human *Stat3* | Forward  Reverse | 5’-GGCATTCGGGAAGTATTGTCG-3’  5’-GGTAGGCGCCTCAGTCGTATC-3’ |
| Human *RELA* | Forward  Reverse | 5′-CCAGACCAACAACAACCCCT-3′  5′-TCACTCGGCAGATCTTGAGC-3′; |
| Human *Smad2* | Forward  Reverse | 5’-GATCCTAACAGAACTTCCGCC-3’  5’-CACTTGTTTCTCCATCTTCACTG-3’ |
| Human *Smad3* | Forward  Reverse | 5’-ACGTCAACACCAAGTGCATC-3’  5’-TAGGGATTCACGCAGACCTC-3’ |
| Human *TGFB* | Forward  Reverse | 5’-GGGACTATCCACCTGCAAGA-3’  5’-CCTCCTTGGCGTAGTAGTCG-3’ |
| Human *TGFBR1* | Forward  Reverse | 5’-GCAGAGCTGTGAAGCCTTGAGA-3’  5’-TGCCTTCCTGTTGACTGAGTTG-3’ |
| Human *CD279* | Forward  Reverse | 5’-CGTGGCCTATCCACTCCTCA-3’  5’-ATCCCTTGTCCCAGCCACTC-3’ |
| Human *CD25* | Forward  Reverse | 5’-GGAGACAGAGGAAGAGTAGAAG-3’  5’-AGGCAAGCACAACGGATG-3’ |
| Human *CD69* | Forward  Reverse | 5’-TCTCAATGCCATCAGACA-3’  5’-GACAGCAAGAGTAGCACC-3’ |
| Human *GAPDH* | Forward  Reverse | 5’- TAAAGGGCATCCTGGGCTACACT -3’  5’- TTACTCCTTGGAGGCCATGTAGG -3’ |

**Supplemental Table 1. Sequences of forward (F) and reverse (R) primers used for RT-PCR amplification.**

**Supplemental Table 2. Sequences of forward (F) and reverse (R) primers used for mutagenesis.**

| Target Residue | Primer | Sequences |
| --- | --- | --- |
| S83A | Forward  Reverse | 5’- cgagaagtccaccgcgtcttgaagcagcc -3’  5’- ggctgcttcaagacgcggtggacttctcg -3’ |
| T327A | Forward  Reverse | 5’- catccacttcacaggcgagtgactgcacctg -3’  5’- caggtgcagtcactcgcctgtgaagtggatg -3’ |
| T336A | Forward  Reverse | 5’- ccagggactcgttagcgcctttaagggcatc-3’  5’- gatgcccttaaaggcgctaacgagtccctgg -3’ |
| S339A | Forward  Reverse | 5’- ggcgctccagggcctcgttagtgcc -3’  5’- ggcactaacgaggccctggagcgcc -3’ |
| S459A | Forward  Reverse | 5’- gtcatcgtgatgctgagcagtctcattgatcacct -3’  5’- aggtgatcaatgagactgctcagcatcacgatgac-3’ |
| S83E | Forward  Reverse | 5’- cagcgagaagtccacctcgtcttgaagcagccgc -3  5’- gcggctgcttcaagacgaggtggacttctcgctg -3’ |
| T327E | Forward  Reverse | 5’- taagggcatccacttcacactcgagtgactgcacctgtctc -3’  5’- gagacaggtgcagtcactcgagtgtgaagtggatgccctta -3’ |
| S339E | Forward  Reverse | 5'-acgcatctggcgctccagctcctcgttagtgcctttaag-3'  5'-cttaaaggcactaacgaggagctggagcgccagatgcgt-3' |

**Supplemental Table 3. Cox regression analysis for survival of lung adenocarcinoma (LUAD) patients expressing PLK1 and VIM of KM plot used in figure 1c.**

| Endpoint | Gene expression | Number of patients (n) | Hazard ratio (HR) | 95% Confidential interval (CI) |
| --- | --- | --- | --- | --- |
| Overall Survival (OS)  n=660 | VIM^Hi^/PLK1^Hi^ | 48 | 2.244 | 1.480 - 3.403 |
|  | VIM^Hi^/PLK1^Lo^ | 147 | 1.238 | 0.899 - 1.706 |
|  | VIM^Lo^/PLK1^Hi^ | 147 | 1.469 | 1.081 - 1.996 |
|  | VIM^Lo^/PLK1^Lo^ | 318 | - | - |
| Relapse-Free Progression  (RFP)  n=383 | VIM^Hi^/PLK1^Hi^ | 30 | 3.071 | 1.631 - 5.779 |
|  | VIM^Hi^/PLK1^Lo^ | 84 | 1.396 | 0.819 - 2.381 |
|  | VIM^Lo^/PLK1^Hi^ | 84 | 1.439 | 0.851 - 2.433 |
|  | VIM^Lo^/PLK1^Lo^ | 185 | - | - |

**Supplemental Table 4. Cox regression analysis for overall survival of LUAD patients expressing PLK1 and VIM of KM plot used in figure 1d.**

| Tumor Stage | Gene expression | Number of patients (n) | Hazard ratio (HR) | 95% Confidential interval (CI) |
| --- | --- | --- | --- | --- |
| Stage 1  (n=360) | VIM^Hi^/PLK1^Hi^ | 84 | 2.090 | 1.202 - 3.634 |
|  | VIM^Hi^/PLK1^Lo^ | 96 | 1.045 | 0.566 - 1.930 |
|  | VIM^Lo^/PLK1^Hi^ | 96 | 1.303 | 0.734 - 2.314 |
|  | VIM^Lo^/PLK1^Lo^ | 84 | - | - |
| Stage 2  (n=132) | VIM^Hi^/PLK1^Hi^ | 27 | 1.431 | 0.693 0 2.952 |
|  | VIM^Hi^/PLK1^Lo^ | 38 | 0.762 | 0.377 - 1.543 |
|  | VIM^Lo^/PLK1^Hi^ | 38 | 1.118 | 0.560 – 2.231 |
|  | VIM^Lo^/PLK1^Lo^ | 29 | - | - |

**Supplemental Table 5. Types of lung cancer cell lines used in Figure 1e.**

| Lung cell line | Origin | Tumor Stage |
| --- | --- | --- |
| A549 | Adenocarcinoma | Primary lung cancer |
| NCI-H522 | Adenocarcinoma | Stage 2 |
| NCI-H1944 | Adenocarcinoma | Stage 3B |
| NCI-H358 | Adenocarcinoma | Metastatic lung cancer |
| NCI-H322 | Adenocarcinoma | Metastatic lung cancer |
| NCI-H2122 | Adenocarcinoma | Stage 4 |

| Tumor Stage | Gene expression | Number of patients (n) | Hazard ratio (HR) | 95% Confidential interval (CI) |
| --- | --- | --- | --- | --- |
| All  (n=631) | VIM^Lo^/ PLK1^Lo^/CD274^Lo^ | 106 | - | - |
|  | VIM^Lo^/ PLK1^Lo^/CD274^Hi^ | 44 | 2.378 | 1.326 - 4.263 |
|  | VIM^Lo^/PLK1^Hi^/CD274^Lo^ | 92 | 2.311 | 1.420 - 3.761 |
|  | VIM^Lo^/PLK1^Hi^/CD274^Hi^ | 75 | 2.125 | 1.266 - 3.568 |
|  | VIM^Hi^/PLK1^Lo^/CD274^Lo^ | 74 | 1.396 | 0.791 - 2.466 |
|  | VIM^Hi^/PLK1^Lo^/CD274^Hi^ | 93 | 1.555 | 0.926 - 2.610 |
|  | VIM^Hi^/PLK1^Hi^/CD274^Lo^ | 45 | 1.999 | 0.791 - 2.466 |
|  | VIM^Hi^/PLK1^Hi^/CD274^Hi^ | 102 | 2.798 | 1.746 - 4.484 |
| Stage 1  (n=346) | VIM^Lo^/ PLK1^Lo^/CD274^Lo^ | 56 | - | - |
|  | VIM^Lo^/ PLK1^Lo^/CD274^Hi^ | 26 | 1.167 | 0.466 - 2.927 |
|  | VIM^Lo^/PLK1^Hi^/CD274^Lo^ | 46 | 1.649 | 0.793 - 3.429 |
|  | VIM^Lo^/PLK1^Hi^/CD274^Hi^ | 45 | 0.958 | 0.420 - 2.184 |
|  | VIM^Hi^/PLK1^Lo^/CD274^Lo^ | 46 | 0.945 | 0.403 - 2.214 |
|  | VIM^Hi^/PLK1^Lo^/CD274^Hi^ | 45 | 0.979 | 0.418 - 2.292 |
|  | VIM^Hi^/PLK1^Hi^/CD274^Lo^ | 25 | 1.300 | 0.518 - 3.261 |
|  | VIM^Hi^/PLK1^Hi^/CD274^Hi^ | 57 | 2.527 | 1.297 - 4.923 |
| Stage 2  (n=118) | VIM^Lo^/ PLK1^Lo^/CD274^Lo^ | 17 | - | - |
|  | VIM^Lo^/ PLK1^Lo^/CD274^Hi^ | 10 | 0.932 | 0.315 - 2.757 |
|  | VIM^Lo^/PLK1^Hi^/CD274^Lo^ | 19 | 1.375 | 0.537 - 3.519 |
|  | VIM^Lo^/PLK1^Hi^/CD274^Hi^ | 14 | 0.916 | 0.317 - 2.651 |
|  | VIM^Hi^/PLK1^Lo^/CD274^Lo^ | 17 | 0.636 | 0.220 - 1.843 |
|  | VIM^Hi^/PLK1^Lo^/CD274^Hi^ | 16 | 0.719 | 0.257 - 2.008 |
|  | VIM^Hi^/PLK1^Hi^/CD274^Lo^ | 7 | 1.755 | 0.522 - 5.898 |
|  | VIM^Hi^/PLK1^Hi^/CD274^Hi^ | 18 | 1.419 | 0.558 - 3.611 |

**Supplemental Table 6. Cox regression analysis for overall survival of LUAD patients expressing PLK1, VIM, and CD274 of KM plot used in figure 8a.**
