# Supplementary figures and images for: PLK1/vimentin signaling facilitates immune escape by recruiting Smad2/3 to PD-L1 promoter in metastatic lung adenocarcinoma
Source: Cell Death Differ. 2021 May 7;28(9):2745–64. doi: 10.1038/s41418-021-00781-4 (PMC8408167; doi:10.1038/s41418-021-00781-4)

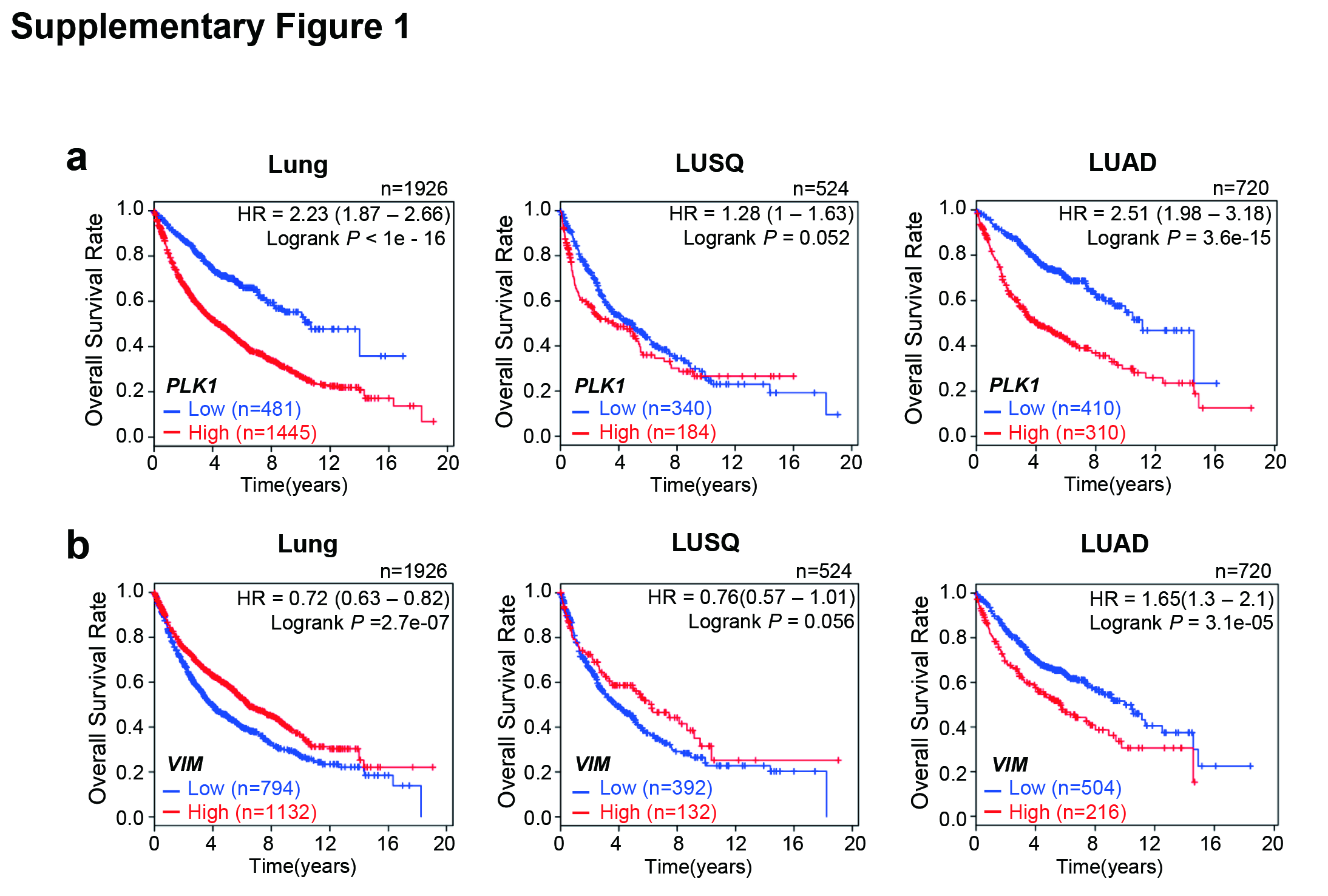

Supplement: Supplementary file 3 — Supplementary Figure 1. [file 41418_2021_781_MOESM3_ESM.tif]

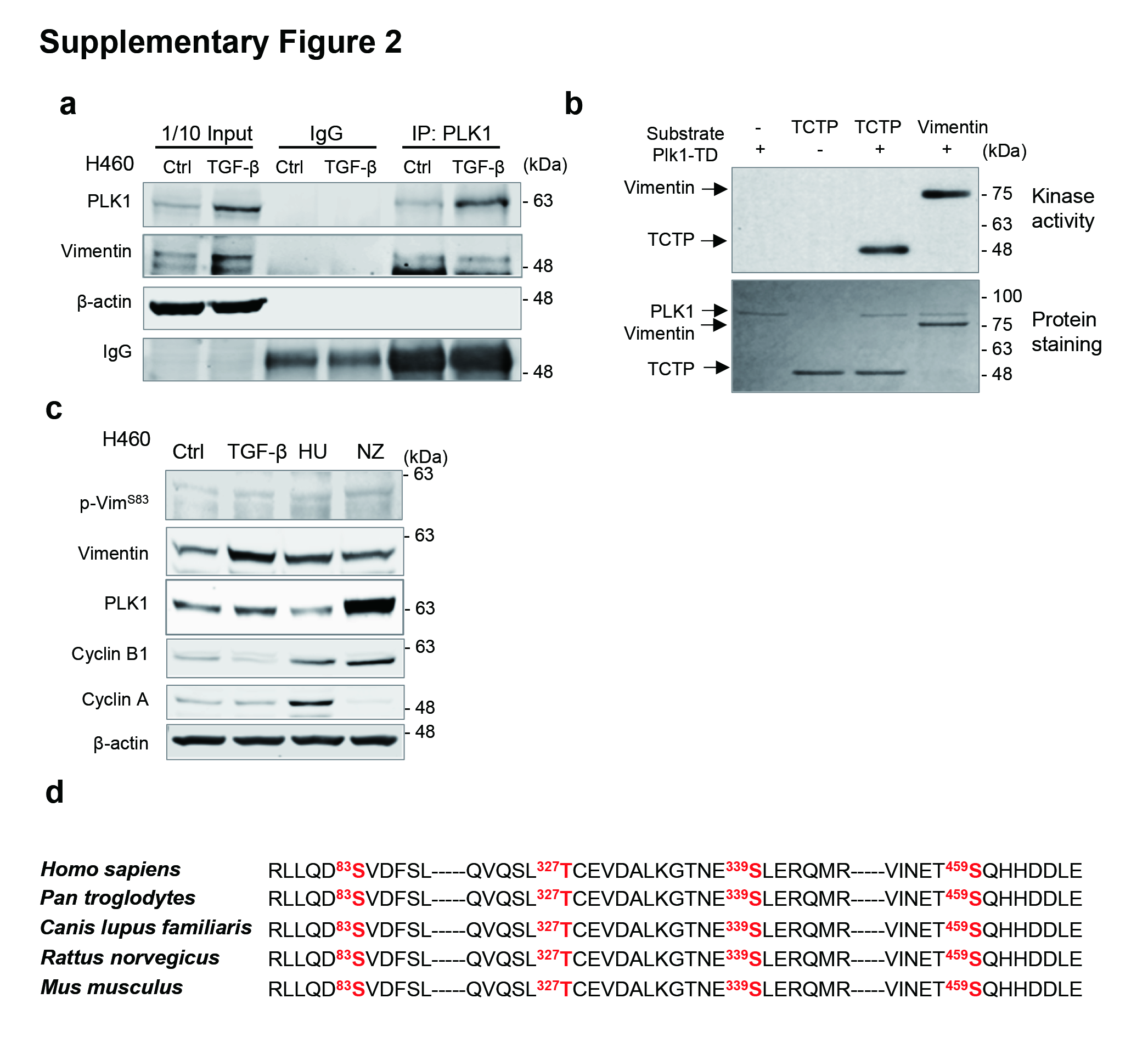

Supplement: Supplementary file 4 — Supplementary Figure 2. [file 41418_2021_781_MOESM4_ESM.tif]

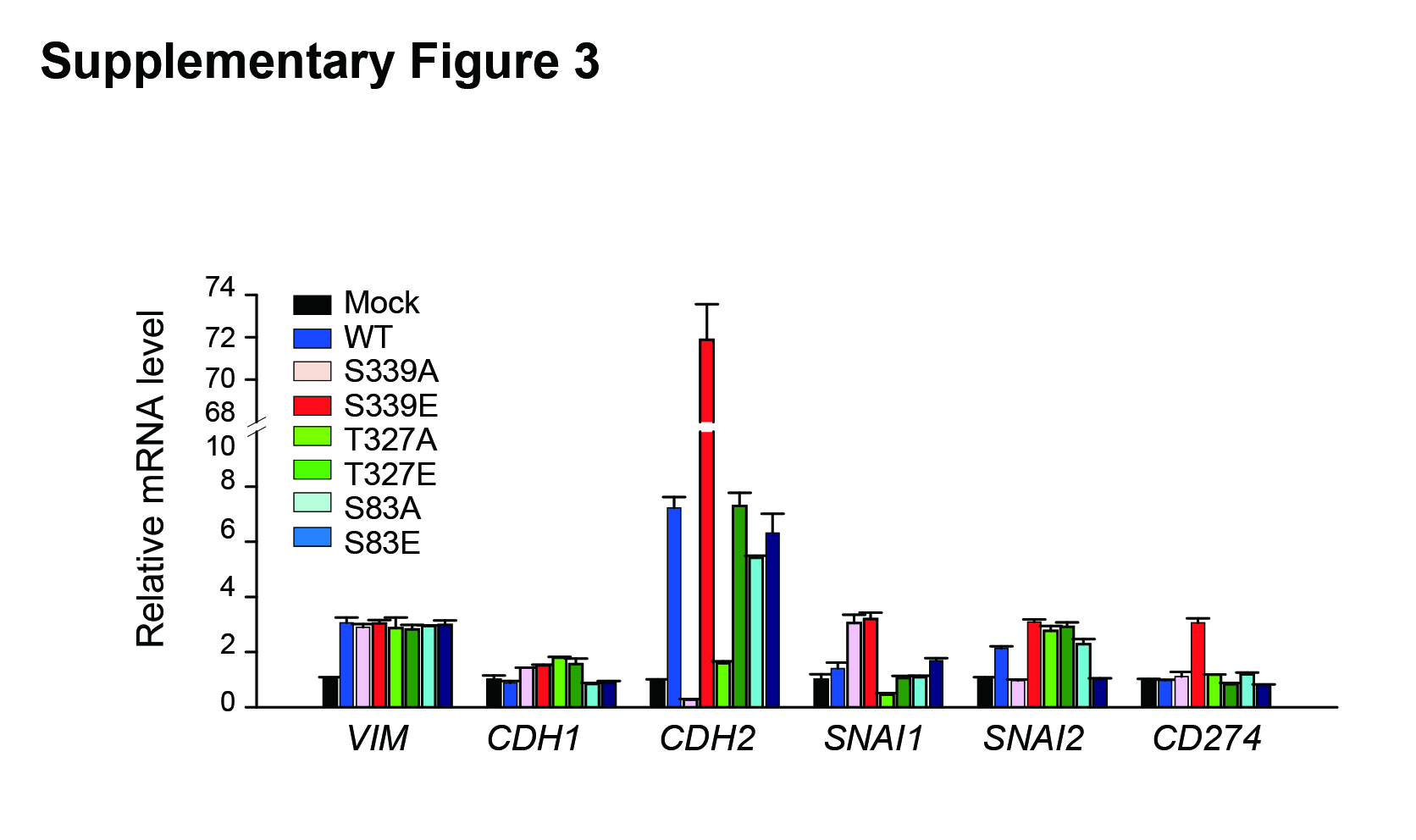

Supplement: Supplementary file 5 — Supplementary Figure 3. [file 41418_2021_781_MOESM5_ESM.tif]

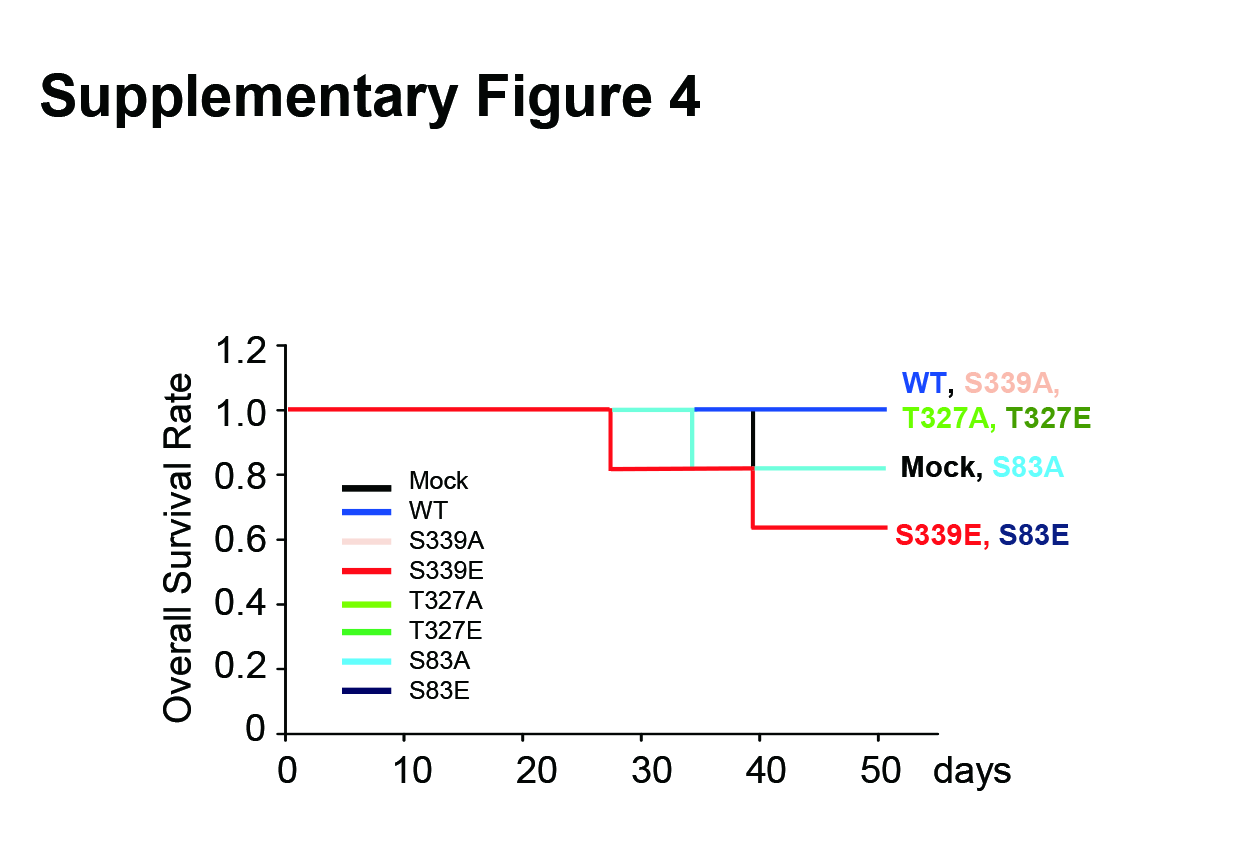

Supplement: Supplementary file 6 — Supplementary Figure 4. [file 41418_2021_781_MOESM6_ESM.tif]

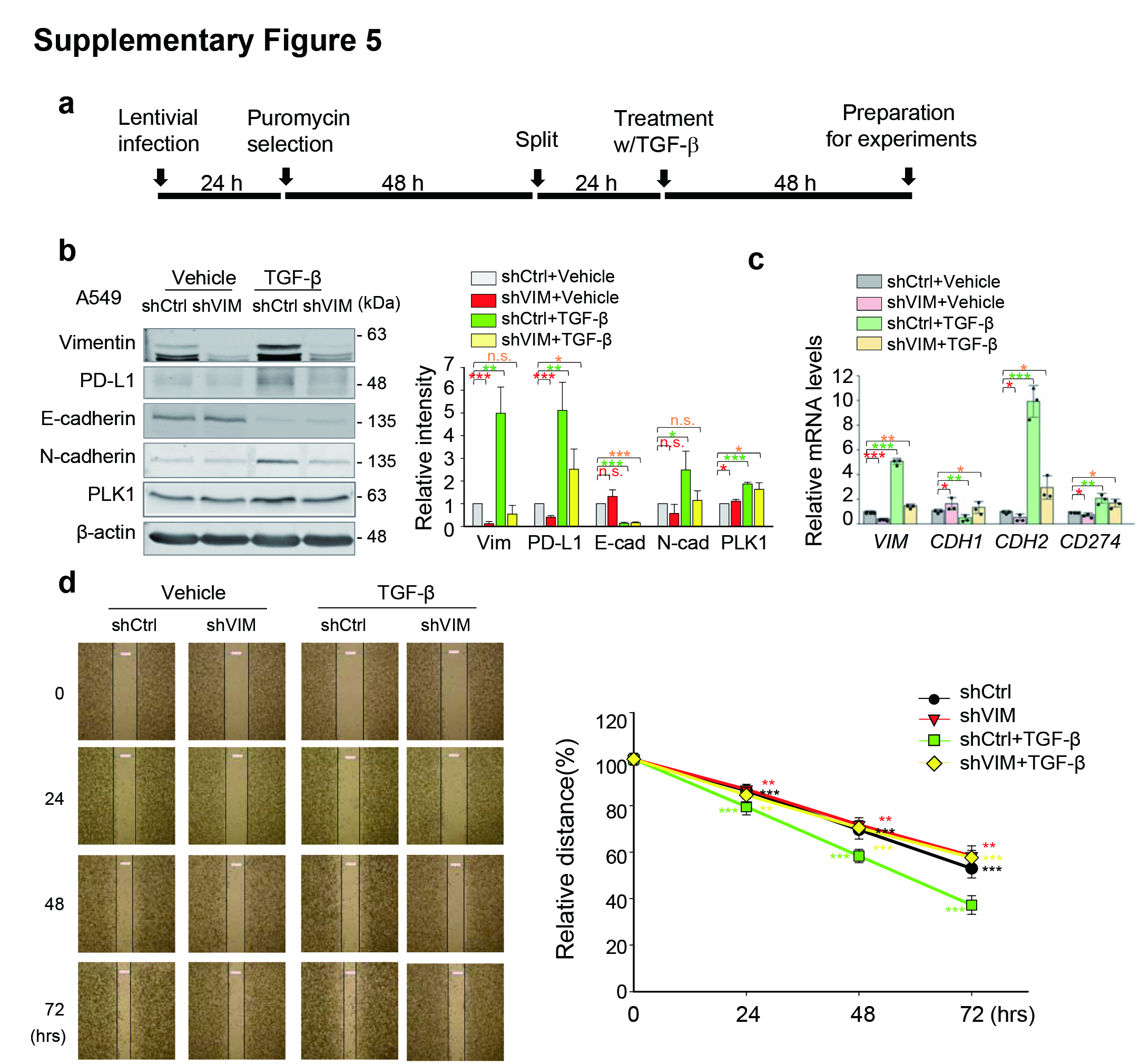

Supplement: Supplementary file 7 — Supplementary Figure 5. [file 41418_2021_781_MOESM7_ESM.tif]

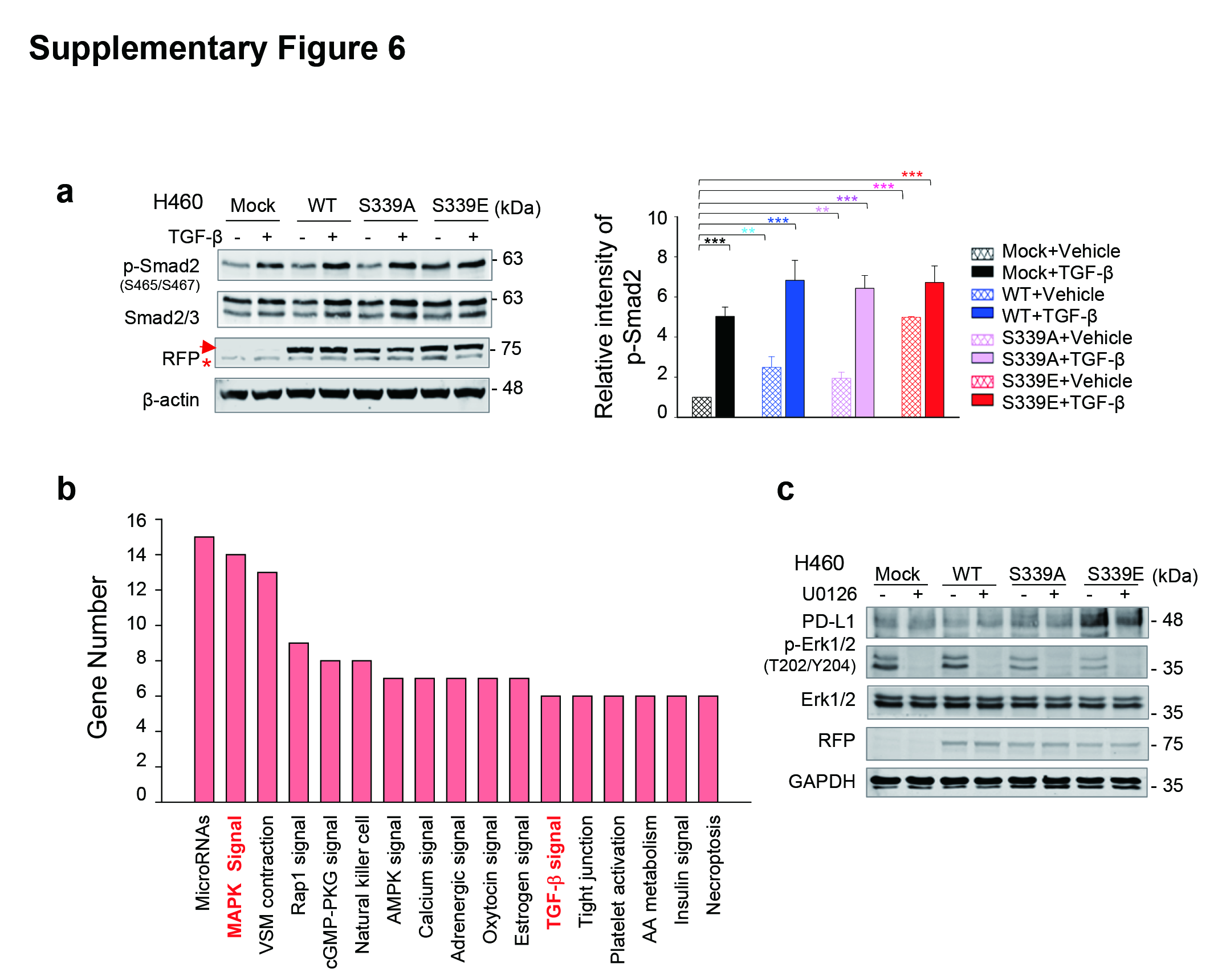

Supplement: Supplementary file 8 — Supplementary Figure 6. [file 41418_2021_781_MOESM8_ESM.tif]

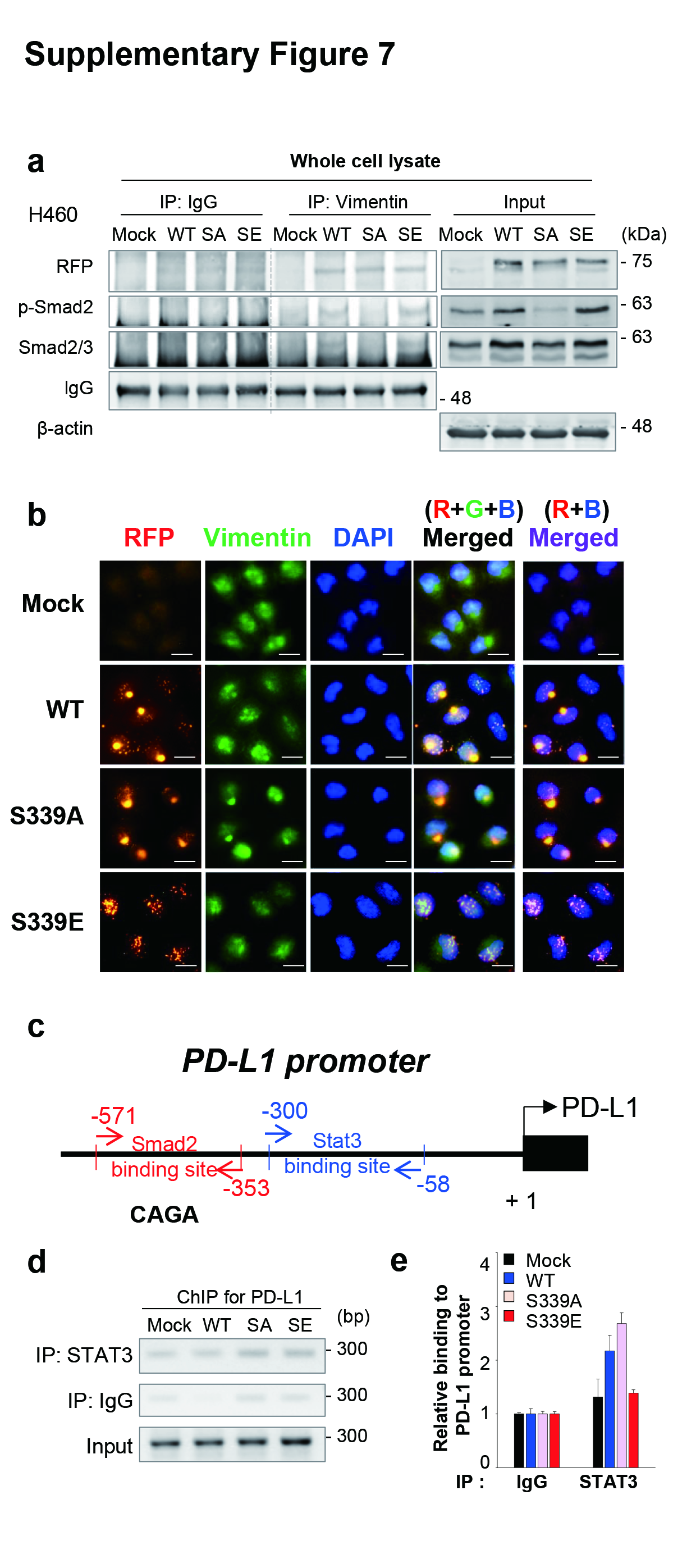

Supplement: Supplementary file 9 — Supplementary Figure 7. [file 41418_2021_781_MOESM9_ESM.tif]

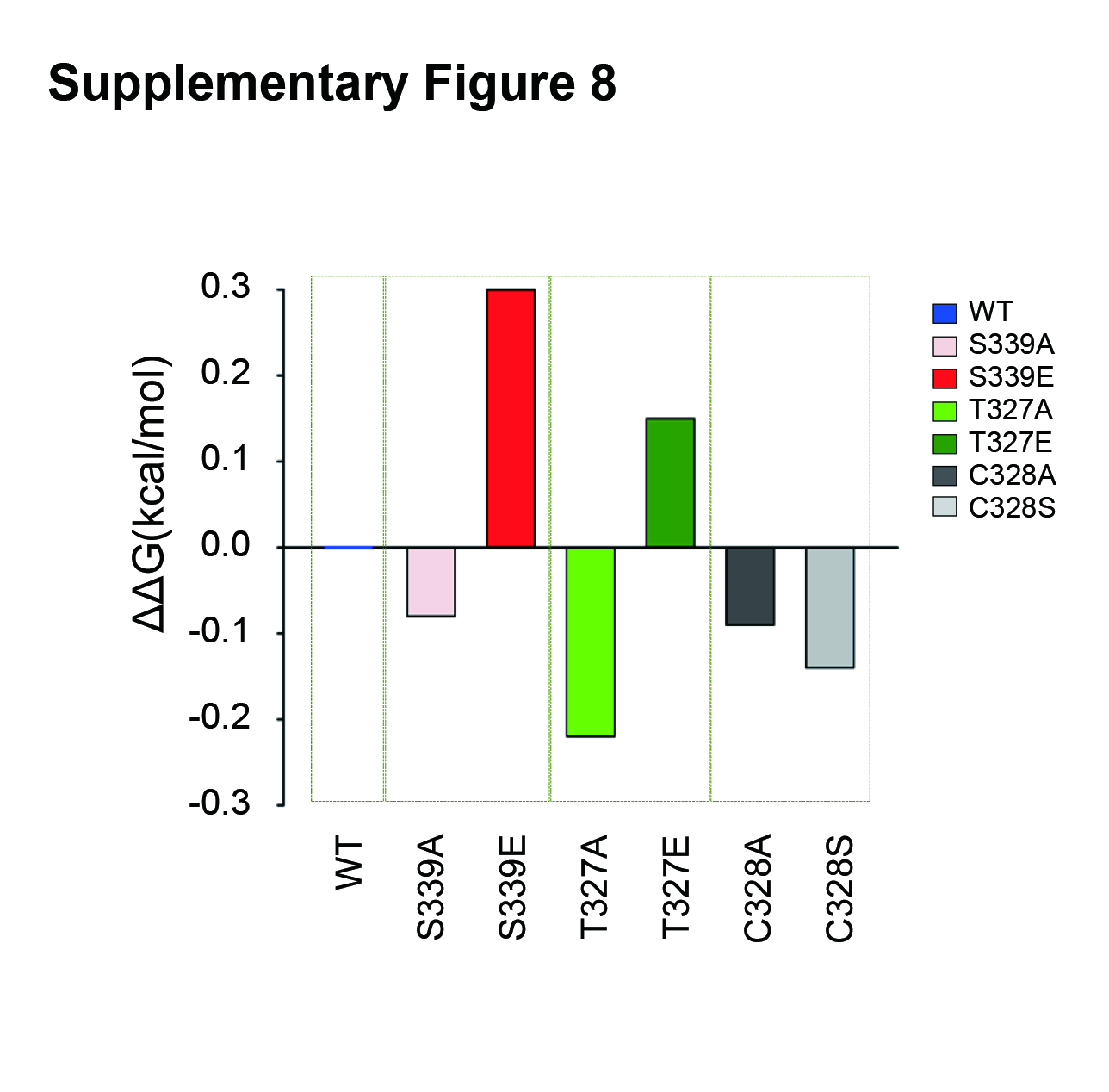

Supplement: Supplementary file 10 — Supplementary Figure 8. [file 41418_2021_781_MOESM10_ESM.tif]

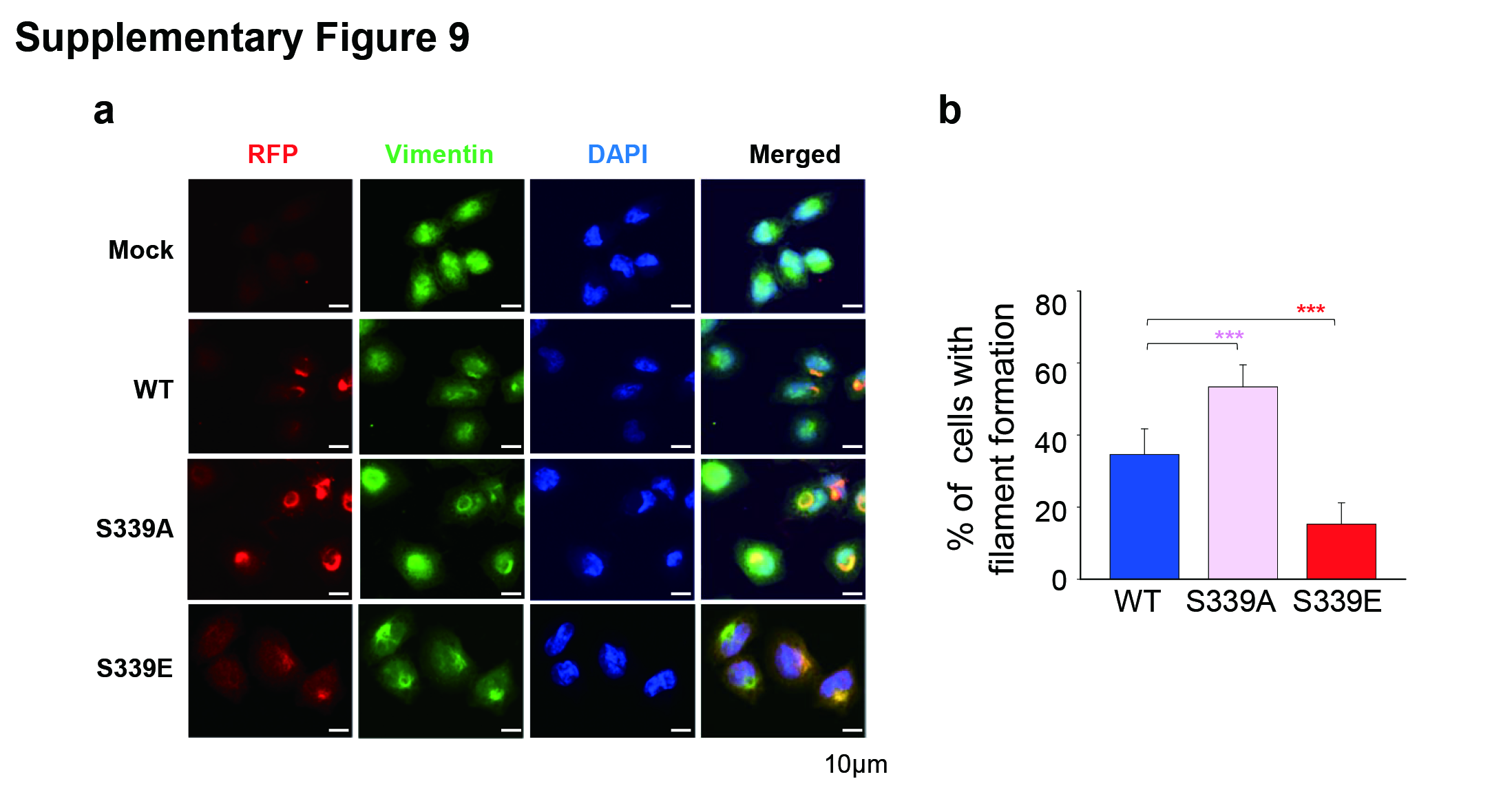

Supplement: Supplementary file 11 — Supplementary Figure 9. [file 41418_2021_781_MOESM11_ESM.tif]

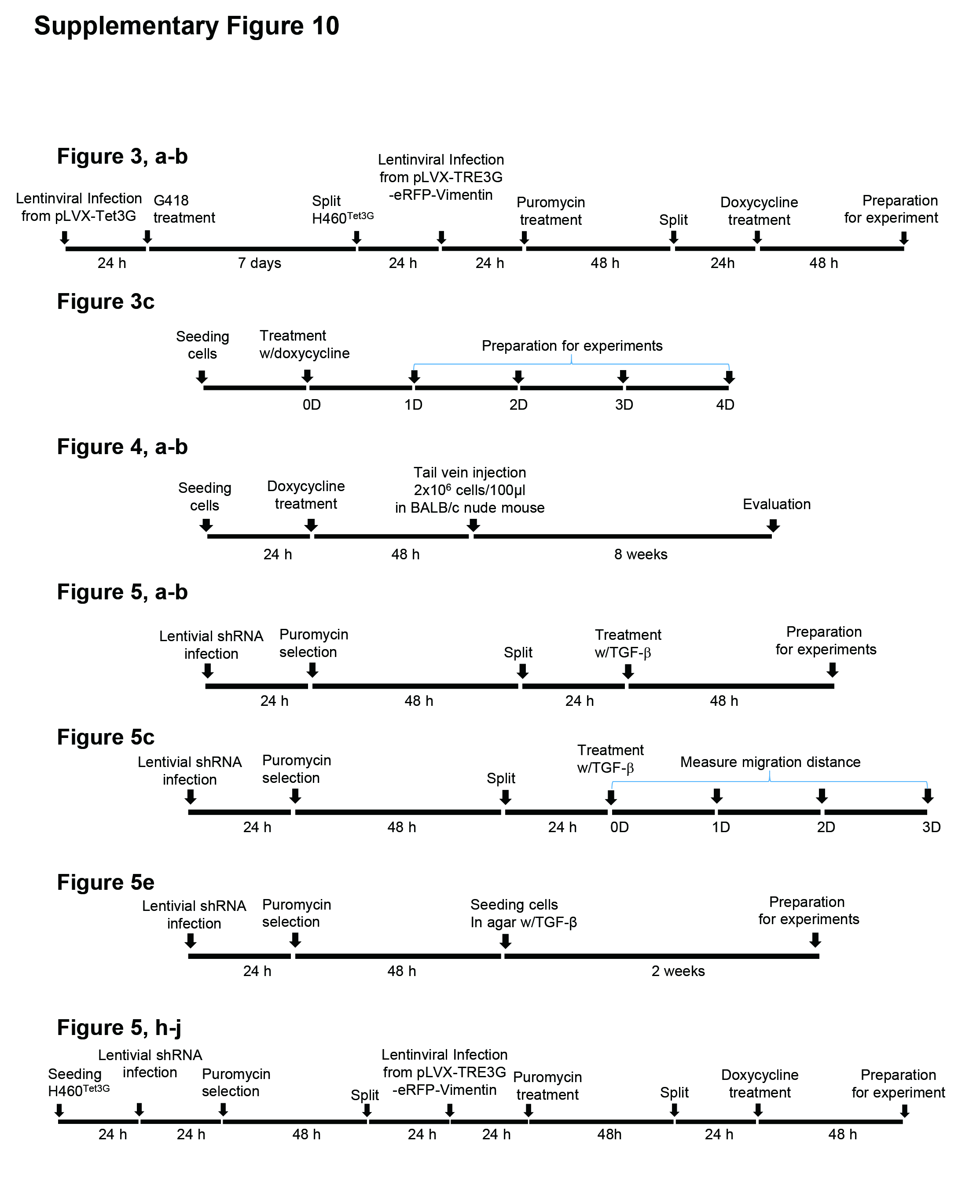

Supplement: Supplementary file 12 — Supplementary Figure 10. [file 41418_2021_781_MOESM12_ESM.tif]
